# Engineered microvesicle-mediated multimodal therapy resolves ulcerative colitis via synergistic immunoregulation, barrier restoration, and microbiota remodeling

**DOI:** 10.1016/j.mtbio.2026.103623

**Published:** 2026-08-31

**Authors:** Cuihua Qi, Chaoyue Liang, Sha Wang, Hui Zhang, Han Wei, Xiaojun He, Weigang Chen

**Affiliations:** a Department of Gastroenterology, The First Affiliated Hospital of Shihezi University, Shihezi, 832008, China; b College of Animal Science and Technology, Shihezi University, Shihezi, 832003, China; c Department of Ophthalmology, Peking University First Hospital, Beijing, 100034, China

**Keywords:** Ulcerative colitis, M2 macrophage-derived microvesicles, TLR4-NF-κB signaling, Gut microbiome, Immunotherapy

## Abstract

Ulcerative colitis (UC) represents a major global health challenge characterized by immune dysregulation and disruption of the epithelial barrier, with current therapies demonstrating limited efficacy. Microvesicles (MVs) engineered through alternative macrophage activation possess immunomodulatory and restorative cargo. However, their therapeutic potential for UC and the underlying mechanisms remain largely unexplored. Herein, we demonstrate that these engineered MVs significantly mitigate colitis symptoms, including weight loss, rectal bleeding, and colon shortening, while also reducing histopathological damage and restoring barrier function in UC. They suppress pro-inflammatory cytokines (IL-1β, IL-6, and TNF-α) and upregulate IL-10 by inhibiting the TLR4/MyD88/NF-κB signaling pathway. Furthermore, they reshape gut microbiota, enhance microbial diversity, and rectify dysbiosis. Collectively, our findings establish a multimodal therapy mediated by engineered MVs, which includes the inhibition of inflammatory signaling, repair of the disrupted epithelial barrier and remodeling of dysbiotic microbiota, as a promising strategy for UC, thereby expanding the therapeutic potential of extracellular vesicles for precision management of UC.

## Highlights

• M2-MVs suppress TLR4/MyD88/NF-κB to alleviate colonic inflammation.
• M2-MVs restore tight-junction and mucus barriers in ulcerative colitis.
• M2-MVs remodel gut microbiota and reverse dysbiosis in colitis.
• M2-MVs integrate immunoregulation, barrier repair, and microbiota modulation.
• M2-MVs outperform unpolarized M0-MVs as a cell-free multimodal therapy.

## Graphical abstract

**Figure:**
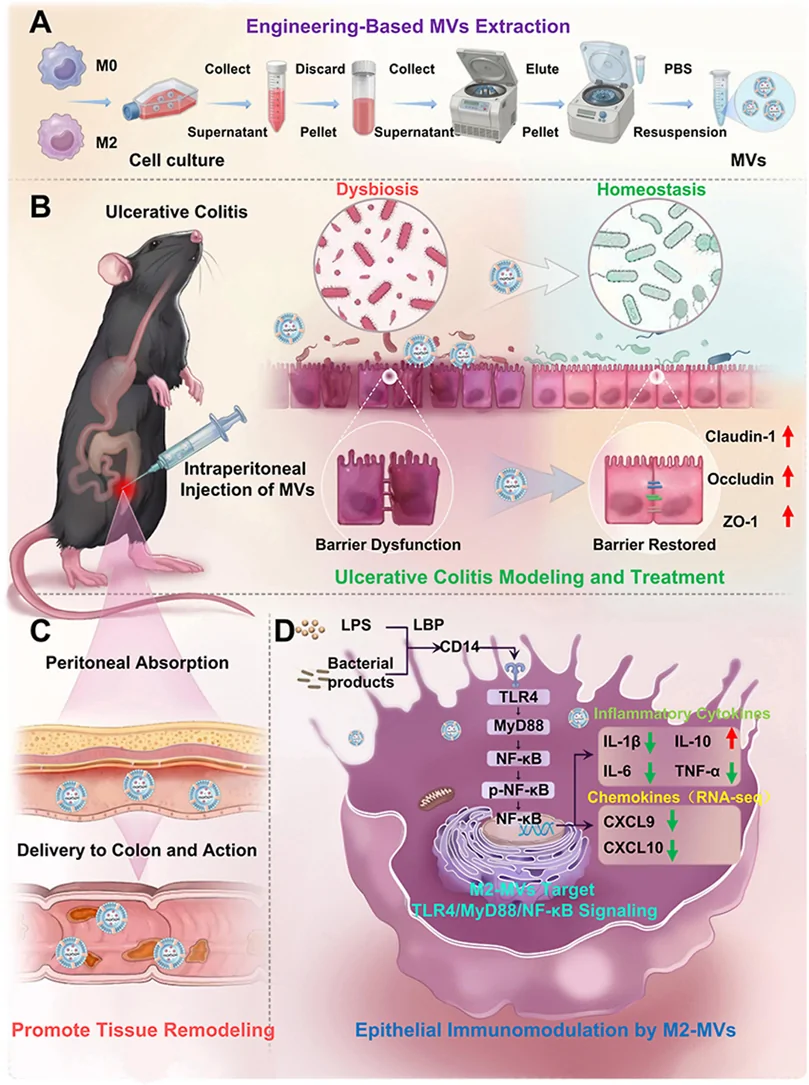

## 1 Introduction

Ulcerative colitis (UC) is a chronic inflammatory disorder driven by a self-perpetuating interaction among mucosal immune dysregulation, epithelial barrier disruption, and gut microbiota dysbiosis [1,2] The pharmacological and surgical treatments currently available to interrupt this chain show low efficacy, refractory disease, and a high incidence of adverse events [3]. Therefore, therapeutic strategies capable of simultaneously restoring intestinal immune and barrier homeostasis are urgently needed.

Intestinal macrophages are the key guardians of mucosal homeostasis, and their functional phenotype resides at the nexus of inflammation and resolution. In the UC microenvironment, macrophages are frequently skewed toward a pro-inflammatory M1 phenotype that aggravates tissue injury [4,5], whereas M2-polarized macrophages promote inflammation resolution and mucosal repair [4,6]. Thus, they are uniquely positioned to address both the inflammatory and barrier-disruption aspects of UC. Although M2 macrophage cell therapy holds promise, it faces several obstacles in clinical translation [7]. For instance, it struggles with poor stabilization *in vivo*, phenotypic reversion to M1 state in inflammatory niche, and safety issues [[8], [9], [10]]. As a result, the therapeutic focus has turned towards the paracrine effectors which primarily mediate these protective functions.

The extracellular vesicles (EVs) made by macrophages may act as an effective cell-free therapeutic platform due to their ability to recreate the functions of their parents [[11], [12], [13]]. The expected efficacious action of MVs *in vivo* is possibly due to the interference of molecular and cellular pathways at the site of pathophysiological events. The protective effects of MVs have been demonstrated in the processes of renal inflammation and rheumatoid arthritis [14]. Although the therapeutic applications of M2-macrophage-derived MVs (M2-MVs) have been explored in other multifactorial pathologies, the signaling pathways via which they exert their therapeutic effect on the inflammation in UC remain poorly defined [[15], [16], [17]].

Recent nanomedicine and biomaterial-based approaches have combined control of intestinal inflammation with epithelial barrier repair and microbiota modulation [18,19]. These advances support integrated therapeutic strategies and provide a rationale for evaluating biologically derived M2-MVs as a cell-free platform capable of coordinating immunoregulation, barrier restoration, and microbiota remodeling.

**Scheme 1:**
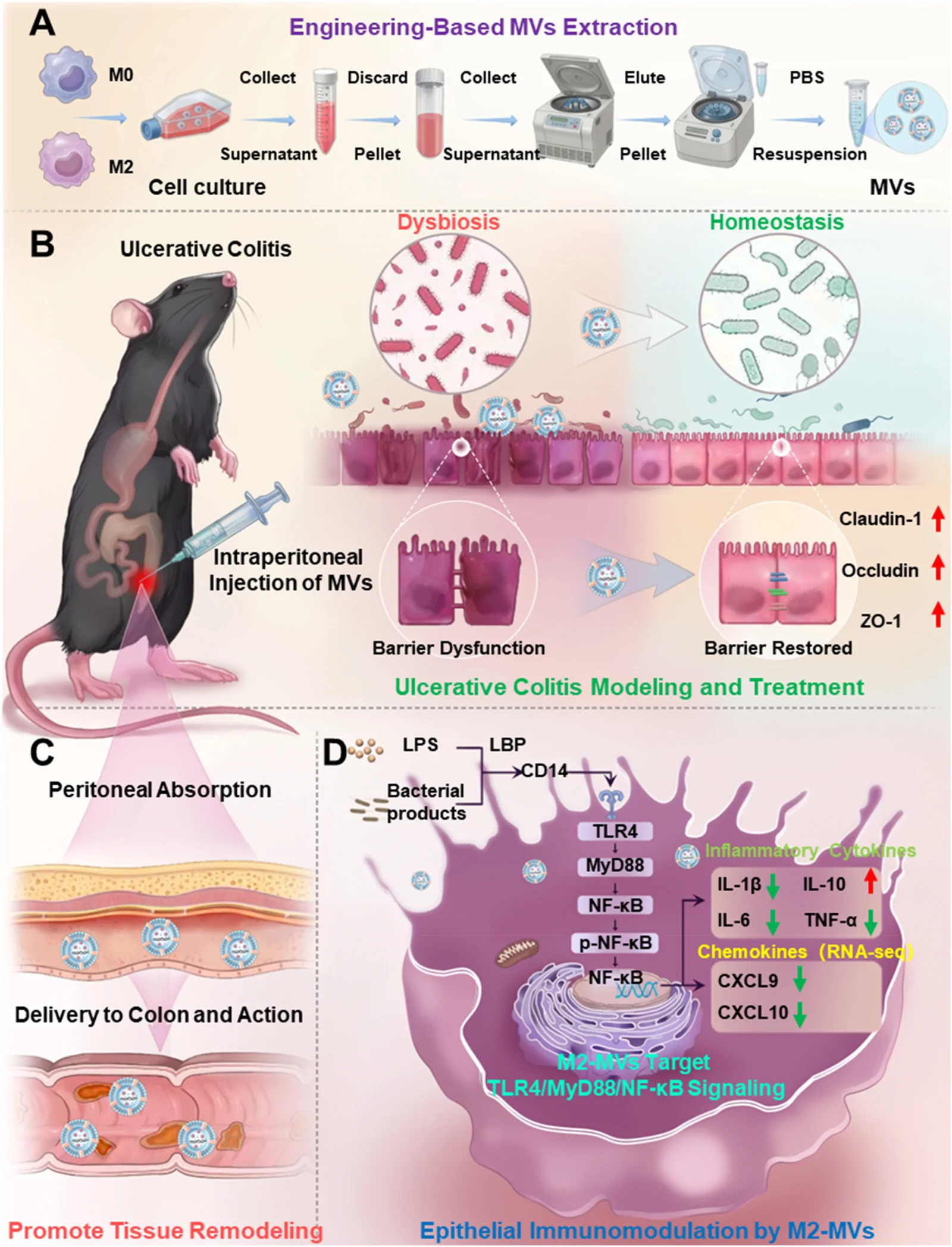
**Isolation and proposed protective mechanisms of M2-MVs in colitis. A,** MVs were enriched from the culture supernatants of M0 or M2 macrophages by differential centrifugation. **B,** In the DSS-induced mouse model, intraperitoneal M2-MV treatment was associated with improved intestinal-barrier markers, including Claudin-1, Occludin, and ZO-1. **C,** The scheme depicts the intraperitoneal route of administration; precise organ distribution, colonic accumulation, retention, and clearance were not determined in this study. **D,** M2-MV treatment was associated with inhibition of TLR4/MyD88/NF-κB signaling, reduced IL-1β, IL-6, TNF-α, CXCL9, and CXCL10, and increased IL-10.

Guided by transcriptomic analysis of colonic epithelial cells treated with M2-MVs, we focused on the TLR4/MyD88/NF-κB pathway, a major innate inflammatory axis that can amplify cytokine production in inflammatory bowel disease (IBD) [[20], [21], [22]]. We thus hypothesized that M2-MVs alleviate the damage on colonic epithelial cells inflicted by this major inflammatory axis, which breaks the cycle of inflammation and barrier damage. Furthermore, this host-directed immunoregulation accompanies the restoration of gut microbial homeostasis [23,24]. Using a dextran sulfate sodium (DSS)-induced colitis model and M0-MVs as a polarization-independent vesicle control, we show that M2-MVs inhibit TLR4/MyD88/NF-κB signaling, restore epithelial tight junctions and the mucus barrier, and remodel gut microbiota composition (Scheme 1). This design tests the polarization-specific activity of M2-MVs and defines an integrated mechanism linking immunoregulation, barrier restoration, and microbiota remodeling in experimental colitis.

## 2 Results and discussion

### 2.1 M2 polarization programs immunoregulatory signatures in MVs

**Fig. 1:**
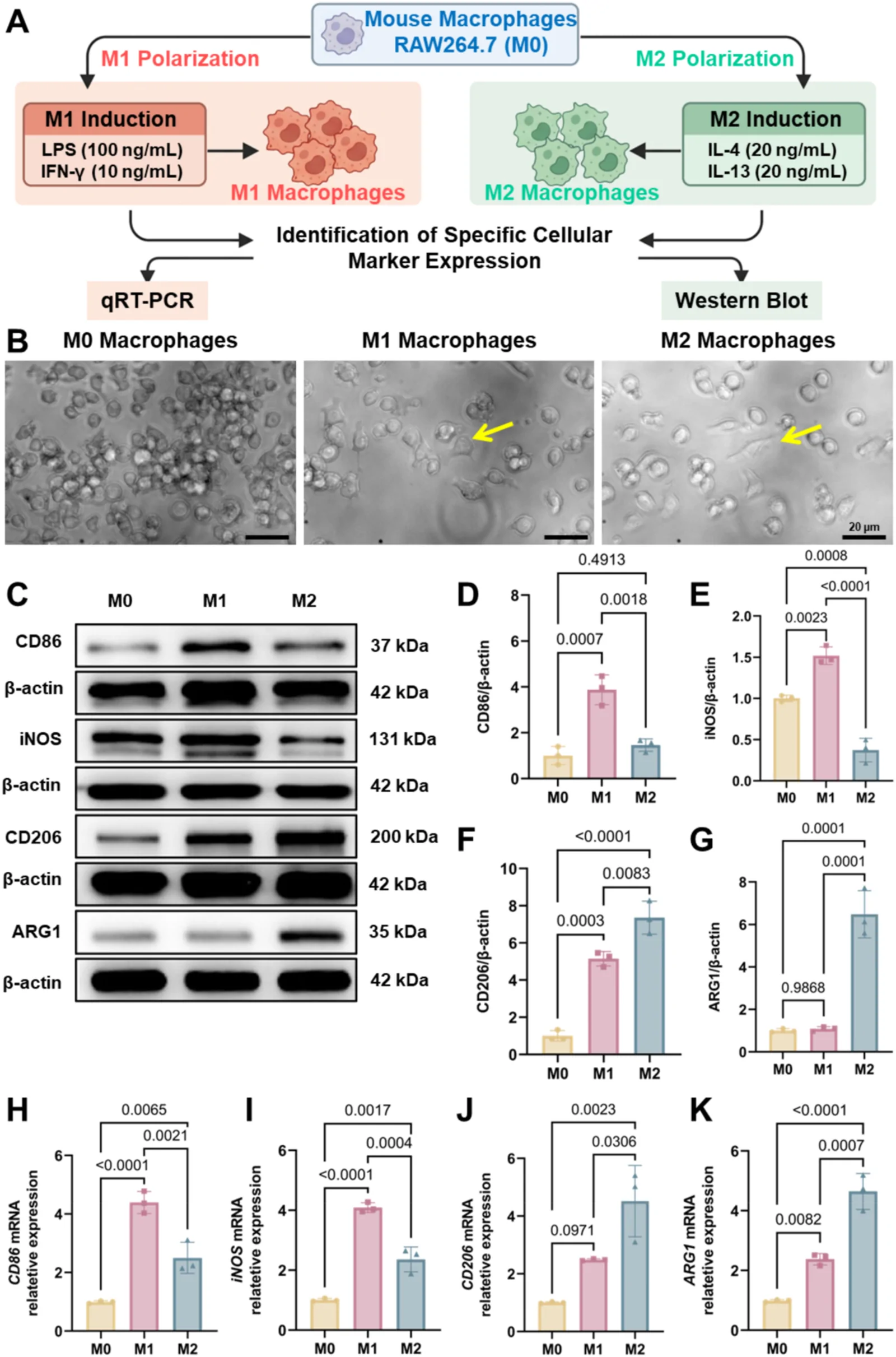
**Identification of macrophage polarization. A,** Schematic illustration of the RAW264.7 macrophage polarization model and detection workflow. **B,** Bright-field images of M0, M1 and M2 macrophages. Yellow arrows indicate polarized cells. **C,** Western blot analysis of polarization marker proteins in cells from each group. **D-G,** Densitometric quantification of CD86, iNOS, CD206, and ARG1 normalized to β-actin. **H-K,** RT-qPCR analysis of the relative mRNA expression levels of polarization markers in cells from each group. (For interpretation of the references to colour in this figure legend, the reader is referred to the Web version of this article.)

The RAW264.7 macrophage cell line was utilized to obtain macrophage models with defined polarization states. Cells that are unstimulated are resting (M0) macrophages. Two established cytokine regimens, i.e., lipopolysaccharide (LPS) plus IFN-γ for classical M1 activation, and IL-4 plus IL-13 for alternative M2 activation (Fig. 1A) were used for polarization. When we examined the morphology of the three groups of cells through microscopy, we found that M1-polarized macrophages are rounded, flattened and spread out in shape [24]. Meanwhile, M2-polarized cells are elongated in shape and mostly spindle-shaped and have protrusions (Fig. 1B). Earlier studies have correlated macrophage functional programming with cell morphology. Indication of the functional state of cells through morphological analysis of cells polarized using these two protocols. We conducted western blotting and RT-qPCR for lineage-defining markers to authenticate molecular polarization state. Western blot analysis exhibited elevated expression of the T-cell costimulatory molecule CD86 and iNOS in M1-polarized macrophages as compared with M0 cells. Conversely, M2-polarized macrophages showed clear up-regulation of CD206 and arginase-1, as shown in Fig. 1C–G. The protein-level alterations described above was reflected at the transcriptional level: selective induction of iNOS and CD86 transcripts in M1 cells and selective enrichment of Arg1 and CD206 transcripts in M2 cells as shown by RT–qPCR (Fig. 1H–K). The functional significance of the co-expression of iNOS and Arg1 was determined within the context of intestinal inflammation. The same substrate L-arginine is competed for by both these enzymes, greatly impacting the macrophage effector functions. The nitric oxide and reactive nitrogen species made by iNOS cause damage to the intestinal epithelium and disruption of tight junctions, which can activate a pro-inflammatory cascade typical of UC [25]. Arg1, on the other hand, directs L-arginine towards the production of ornithine, which produces polyamines or proline that support epithelial cell growth and deposition of collagen and tissue damage or facilitate barrier repair in UC [26]. UC is characterized by persistent inflammation driven by M1 macrophages and damage to the mucosa that prevents healing. The balance between iNOS and Arg1 is what determines whether macrophages drive tissue damage or facilitate barrier repair in UC [27]. In M2-polarized cells, the significantly greater expression of Arg1 compared with iNOS indicates that M2 macrophages and their secreted products have an inherent tissue-protective and repair-promoting function.

This differential signature not only pertains to specific markers but also reflects underlying functional divergence that is directly relevant to UC pathogenesis. The process of endocytosis through CD206 helps in removing injury-related molecular patterns along with bacterial antigens. In general, this removal helps in reducing TLR-driven inflammation. The overexpression of CD86 in M1 macrophages indicates their higher ability to provide costimulatory signals to effector T cells and influence the T-cell response in inflammatory bowel disease. M1 and M2 macrophages can be assigned to respectively pro-inflammatory and anti-inflammatory intestinal microenvironments as per these molecules. We argue that this functional dichotomy is transferred to derived MVs and can explain the different therapeutic efficacy we observed in colitis model. The polarization protocol was validated to be robust as mRNA and protein expression patterns exhibit high correlation. As such, MVs secreted from these polarized macrophages bear functional cargo that reflects a stable commitment to a lineage phenotype, not a transient activation. After obtaining M1 and M2 macrophage populations with different immunoregulatory profiles (pro-inflammatory *vs.* pro-resolving/repair), we isolated and characterized their MVs. The results indicated that MVs derived from M2 exerted strong protective effect against DSS-induced colitis while MVs derived from M0 had a weak or no effect. The therapeutic potential of macrophage-derived MVs is largely dependent on the polarization state of their parent cells, with M2 polarization imparting anti-inflammatory and tissue-repair functions to these MVs.

### 2.2 M2-MVs are efficiently internalized by colonic epithelial cells

**Fig. 2:**
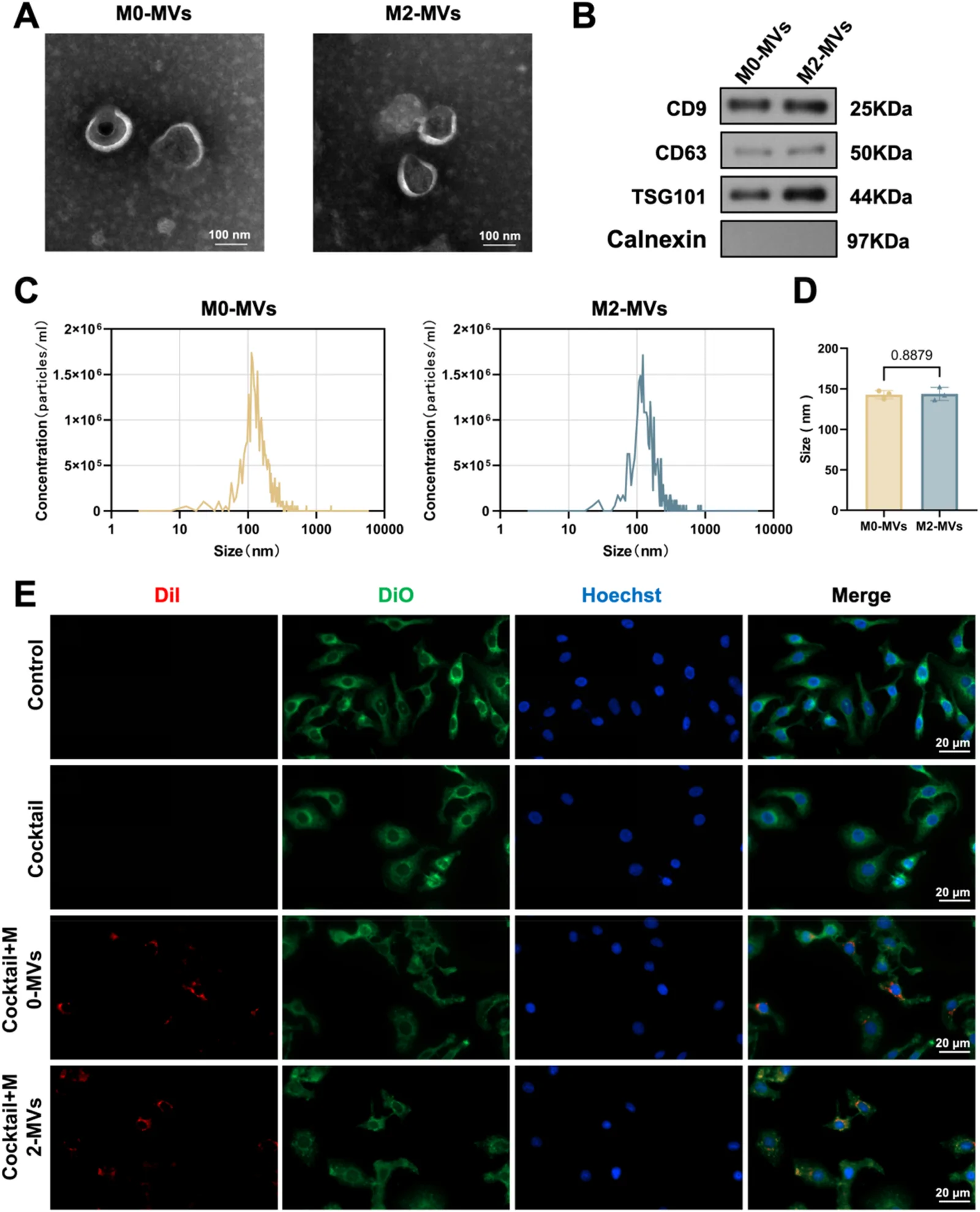
**Isolation, characterization and cellular uptake of macrophage-derived MVs. A,** TEM images of M0-MVs and M2-MVs. **B,** Western blot analysis of the expression of MVs marker proteins, including CD9, CD63, TSG101 and negative marker: Calnexin. **C,** Particle size distribution and concentration of MVs from the two groups were analyzed by nanoparticle tracking analysis (NTA). **D,** Quantitative analysis of the mean particle size of MVs from the two groups. **E,** Fluorescence microscopy of NCM460 cells with DiI-labeled MVs (red); cell membranes were counterstained with DiO (green) and nuclei with Hoechst (blue). (For interpretation of the references to colour in this figure legend, the reader is referred to the Web version of this article.)

To assess the paracrine effectors of M2 macrophages, we first need to identify what EVs they have. The pellets obtained via differential centrifugation were confirmed as MVs-associated particles by TEM, Western blotting and NTA (Fig. S1). Zeta potential analysis further showed that both M0-MVs and M2-MVs exhibited a negative surface charge (Fig. S2). EVs comprise a heterogeneous group of membrane-enclosed particles. Exosomes arise from endosomal multivesicular bodies, whereas MVs (also termed ectosomes) bud from the plasma membrane; size distributions overlap across these classes [27,28]. MVs can be more easily isolated from cell cultures when compared to exosomes. It is also possible that the larger size of these vehicles provides increased loading capacity for bioactive molecules, which can be beneficial to the translational application of cell-free therapies [28,29]. Consequently, differential centrifugation was used to isolate MVs for functional studies. Both the M2-MVs and the M0-MVs are spherical vesicles with well-defined edges and a bilayer membrane structure, as a transmission electron microscopy experiment revealed (Fig. 2A). This implies that the vesicles were formed via direct budding from the plasma membrane. Analysis through western blot additionally affirmed that both MVs populations exhibited the classical extracellular vesicle markers CD9, CD63, TSG101 and negative marker: Calnexin (Fig. 2B), confirming the molecular identity of the purified vesicles.

Upon conducting nanoparticle tracking analysis, it was found that the M2-MVs possessed a size range of 100-1000 nm, with a dominant peak of 137 nm and a relatively narrow distribution (Fig. 2C and D). These observations indicate that the differential centrifugation protocol was effective in enriching a highly pure MVs population. The size profile was substantially different from that of exosomes. This further confirmed the selectivity and reliability of our isolation strategy. The broad size distribution and the greater mean diameter of MVs suggest these vesicles can carry a diverse and abundant repertoire of effector molecules, including proteins, nucleic acids and lipids, and therefore MVs may serve distinct biological functions of intercellular communication.

We subsequently analyzed if MVs could be internalized by colonic epithelial cells, which is necessary for their immunomodulatory as well as barrier-protective activities in situ. CCK-8 assays showed that 15 ng/mL M0-MVs or M2-MVs had no significant effect on NCM460 cell viability and was therefore used in subsequent experiments (Fig. S4). NCM460 cells were incubated with Dil-labeled M0-MVs (or M2-MVs) at a concentration of 15 ng/mL for 12 h and counterstained with Hoechst. Fluorescence imaging of treated control and cytokine-induced inflammatory models revealed strong deep-red signals in the cytoplasmic and perinuclear regions (Fig. 2E). This showed that colonic epithelial cells successfully internalize exogenous MVs. These findings suggest that MVs serve as important paracrine mediators between macrophages and intestinal epithelial cells, and indicate that intestinal epithelial cells are one key cellular target through which M2-MVs exert protective effects in the UC microenvironment [30]. Therefore, we have devised a simple and effective method for obtaining MVs that are highly enriched for MVs and largely depleted of exosomes. According to the method of generating M2-MVs used in this study, the M2-MVs here are pure, structurally intact, and capable of targeting intestinal epithelial cells. This offers an ideal material basis for follow-up *in vivo* and *in vitro* evaluation of M2-MVs protective effects against UC and provides the rationale for clearly choosing the cellular target in future mechanistic studies for intercellular communication.

### 2.3 M2-MVs ameliorate DSS-induced colitis phenotypes and histopathology

**Fig. 3:**
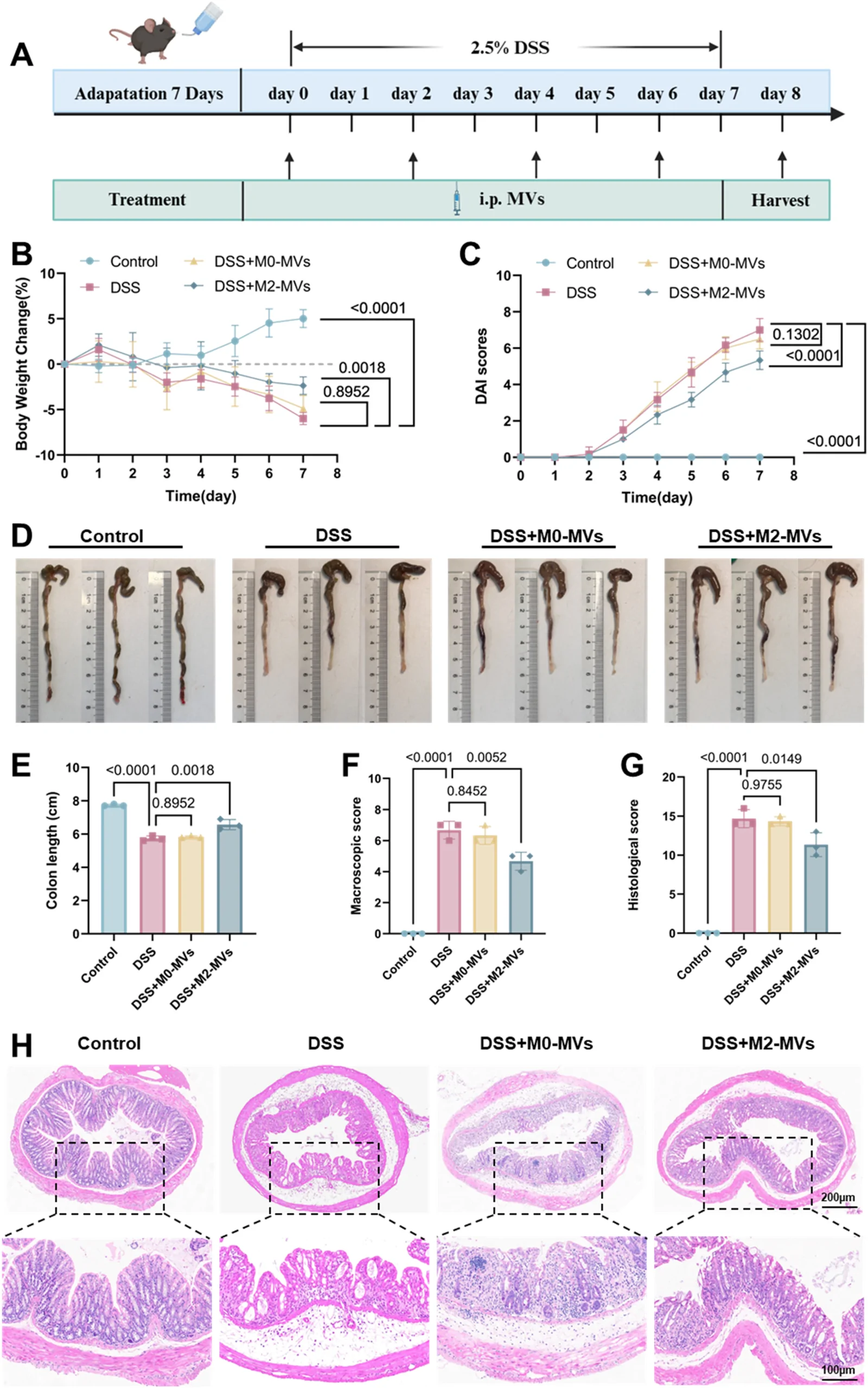
**M2-MVs alleviate DSS-induced colitis in mice. A,** Schematic diagram of the experimental design for DSS-induced acute colitis and MVs intervention. **B,** Body weight change during the experiment. **C,** Disease activity index (DAI) scores. **D,** Representative images of colon length. **E,** Quantitative analysis of colon length. **F,** Gross colonic injury scores. **G,** Histological injury scores. **H,** Representative H&E-stained colon sections.

To evaluate the therapeutic efficacy of MVs from different polarization states of macrophages in UC, MVs from M0 and M2 polarized macrophages were administered in a DSS-induced acute colitis model (Fig. 3A). Both preparations were administered at 50 μg per mouse to permit a consistent comparison. This dose was selected on the basis of published macrophage-derived MVs studies and experimental feasibility; a formal dose-response study was not performed. The DSS challenge induced significant body weight loss, colon shortening, visible congestion and ulceration, and an increase in the disease activity index (DAI) score, which are typical features of experimental colitis. The absence of any haematochezia and the normal stool colour and consistency was seen in the Control group. However, in the DSS group, the colour of the faeces was markedly dark along with obvious haematochezia. This suggests that DSS brought about greater intestinal inflammation and damage to the mucosa. In comparison to the DSS group, M2-MVs treatment markedly improved these faecal abnormalities and the incidence of haematochezia, while no meaningful improvement was observed in the M0-MVs group (Fig. S3). The administration of M2-MVs significantly improved the colitis phenotype induced by DSS. This was confirmed by the recovery of body weight, an increase in colon length, a reduction in macroscopic colonic damage, and a decrease in DAI scores (Fig. 3B–F and Table S1). The consistent protective effects across several different parameters suggest that M2-MVs do not just delay disease onset and/or inhibit a single pathological pathway, but rather coordinate the regulation of multiple disease processes that characterize colitis, such as systemic wasting, colonic tissue remodeling and clinical morbidity. Comparatively, M0-MVs had almost no protective effects, as all parameters were comparable with the DSS group. The differential effect of treatment efficacy of MVs points to their polarization-state dependent function. We found the property of M2 macrophages that induces this treatment efficacy is not transferred onto all macrophage-derived MVs but is acquired through M2-polarization. Macrophages that M2 polarizations exhibit immunomodulatory functions and tissue reparative activity through paracrine effectors enclosed in MVs. The efficacy of the treatment depends on the directed cargo, indicating a biological effect that leads to a change of cellular state.

Histopathological investigation further clarified the extent of protection afforded by M2-MVs. Colonic lesions induced by DSS showed extreme loss of goblet cells and severe disruption of the architecture and area of the crypt. The submucosa was marked by pronounced oedema and extensive infiltration of transmural inflammatory cells (Fig. 3G and H, Table S2). The histological characteristics represent three mechanistically distinct yet interconnected pathological components, including immune-mediated cell recruitment and activation; damage to epithelial stem-cell niches with loss of crypts; and impairment of the secretory lineage and mucus-barrier competence. Treatment with M2-MVs had a coordinated influence on inflammation, crypts and goblets. Following the administration of M2-MVs, there was a significant reduction in inflammatory infiltration. The crypt structures were mostly preserved with more intact morphology. Furthermore, number of goblet cells was restored up to around 50%. M2-MVs are likely not acting on a single immune or epithelial pathway to achieve their *in vivo* mending effects, but rather aim at restoring both compartments simultaneously. This evident from the concurrent recovery of multiple elements. This two-pronged action fits the biology of M2 macrophages, which undertake cytokine secretion for anti-inflammatory activity alongside pro-resolving and tissue-repair activities. Our results indicate that MVs produced by these cells acquire a functional programme from their parental cells that they then relay to target tissues. In contrast, M0‐MVs treatment did not confer any histological benefits, with levels of crypt loss, goblet cell depletion and inflammatory infiltration like those in DSS.

The compositional disparity between M2-and M0-derived MVs further supports the idea that the polarization state determines MVs functional specificity. The immunological and epithelial repair programmes that are necessary for conferring mucosal protection can only be activated by M2-derived MVs. In comparison, M0 macrophages are regarded as non-activated and unpolarized, which suggests that the MVs derived from M0 macrophages might be lacking the anti-inflammatory mediators, pro-resolving factors and barrier-protective molecules enriched by IL-4/IL-13 driven M2. The failure of M0-MVs to exhibit significant activity in any of the phenotypic or histological assays serves as a useful internal negative control. Accordingly, we can exclude the possibility that the activity of our M2-MVs is due to any non-specific property of macrophage MVs. In particular, our negative results are inconsistent with the idea that generic membrane components, or ubiquitous cargoes, account for the protective activity we observe with M2-MVs. These results show that the therapeutic efficacy of M2-MVs depends on the polarization state of their parental cells. M2-derived MVs collectively alleviate the DSS-induced colitis phenotypes and multi-level histopathological damage by concurrent regulation of multiple pathogenic mechanisms, representing the source specificity of MVs function, which is regulated by the polarization status of parent macrophages.

### 2.4 M2-MVs restore colonic barrier by repairing tight junctions and mucus

**Fig. 4:**
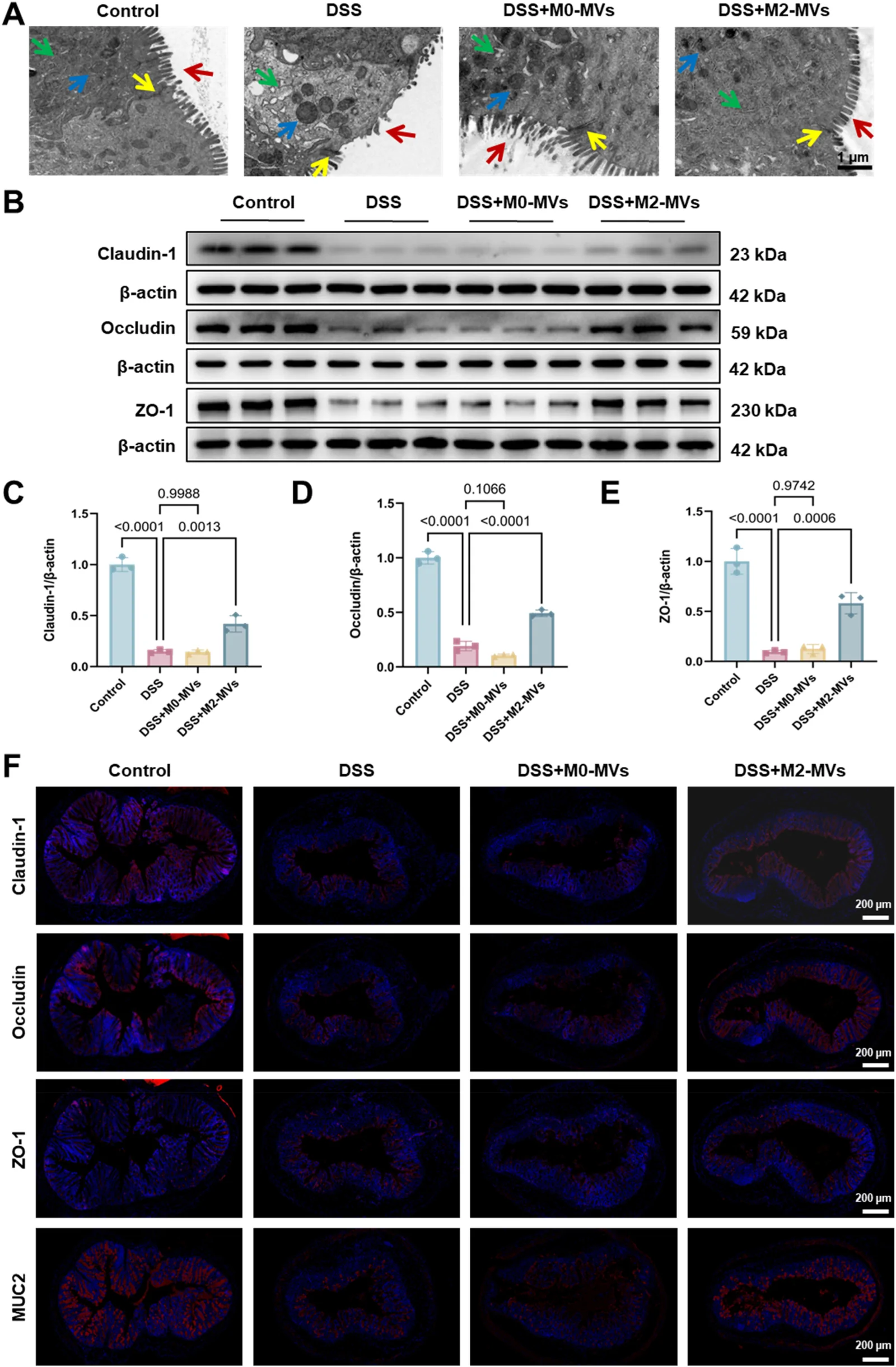
**M2-MVs ameliorate colonic epithelial barrier injury in DSS-treated mice. A,** TEM images of colonic epithelial ultrastructure. Red arrows, microvilli; yellow arrows, tight junctions; blue arrows, indicate mitochondria in the normal group and autolysosomes containing mitochondria in the other groups; green arrows, endoplasmic reticulum. **B-E,** Western blot analysis of the expression of tight junction proteins in colon tissues from each group and the corresponding densitometric quantification. **F,** Immunofluorescence staining of Claudin-1, Occludin, ZO-1, and MUC2 (red) in colon sections. (For interpretation of the references to colour in this figure legend, the reader is referred to the Web version of this article.)

We conducted systematic analyses at the ultrastructural, protein expression, and tissue localization levels to evaluate the restorative effect of M2-MVs on DSS-induced colonic injury [31]. Through transmission electron microscopy analysis, we revealed that DSS-treated mouse colonic epithelia exhibit structural disruption including distortion of tight junction, disintegration of desmosome, widening of intercellular spaces and shortening of microvilli. At the organelle level, the tissues exhibited a decreased content of organelles characterized by sparse or absent cristae, which is consistent with metabolic dysregulation, failure of oxidative phosphorylation, and/or oxidative stress. Additionally, dilated cisternae of the rough endoplasmic reticulum indicate the activation of the unfolded protein response, while an increased number of lysosome-like dense bodies suggests enhanced autophagic or degradative activity [32]. Continuous inflammatory stress causes a failure of epithelial cells to maintain structural and secretory integrity due to the alterations of these organelles. After the M2-MVs intervention, the abnormalities like the intercellular junctional structures and brush-border microvilli were partially restored in morphology and arrangement and organelle injury phenotypes were ablated (Fig. 4A). These findings suggest that M2-MVs not only modulate inflammatory responses but also restore overall homeostasis and epithelial integrity at the organelle level. Proteins associated with the tight junctions are important for determining epithelial barrier function. Western blot analysis demonstrated that the expression of these three proteins was significantly higher upon treatment with M2-MVs versus DSS (Fig. 4B–E). Immunofluorescence staining confirmed that the continuous punctate junctional localization of proteins was disrupted in the DSS group but was fully restored by M2-MVs treatment along the epithelial border. Significantly, the recuperation of these three proteins occurred in a definite order; ZO-1 functions as a scaffolding platform for the tight junction complex, Occludin is a transmembrane protein that regulates paracellular permeability while Claudin-1 functions in the control of barrier selectivity as well as tight junction strand formation. Hence, ZO-1 restoration provides the basis for subsequent Occludin and Claudin-1 reconstruction. The simultaneous upregulation of all three proteins implies that M2-MVs facilitate a significant restoration of the epithelial tight junction architecture through the proper reassembly sequence necessary for functional barrier properties.

We next performed MUC2 immunofluorescence staining to further assess the mucus barrier the first line of defence separating the epithelium from luminal antigens and microbes. MUC2 signals were reduced in the DSS group along with discontinuous mucus layer. M2-MVs treatment, on the other hand, resulted in increased MUC2 and a relatively continuous distribution pattern along the epithelial surface (Fig. 4F and S5). These results imply that M2-MVs not only reduce damage to tight junctions but also shield and rehabilitate the mucus barrier. The integrity of the mucosal layer of the gut is modulated not just by the epithelial secretory capacity but also by gut microbiota. Certain commensal bacteria favour the production and stabilization of mucus, while mucolytic or inflammation-associated ones degrade and thin the mucus. The changes observed in MUC2 continuity after treatment with M2-MVs suggest these M2-MVs strengthen the mucus barrier indirectly, most likely through microbial community remodeling and modulation of microbe-mucus interactions. Further, we test this latter hypothesis through gut microbiota analysis. Taken together, the multi-dimensional evidence reveals that M2-MVs attenuate DSS-induced colonic epithelial barrier injury. M2-macrophages may break this vicious cycle of barrier breakdown and mucosal injury, which allows translocation of luminal antigens and microbe-associated molecules that continuously activate pro-inflammatory signaling pathways. In addition, M2-MVs also initiate hierarchical reinforcements of tight junction proteins, facilitate membrane repair and reduce organelle stress. The potential intestinal mucosal protective activity of M2-MVs could be dependent on their barrier protecting and immuno-modulatory activity.

### 2.5 M2-MVs transcriptionally reprogramme the inflamed colonic epithelium

**Fig. 5:**
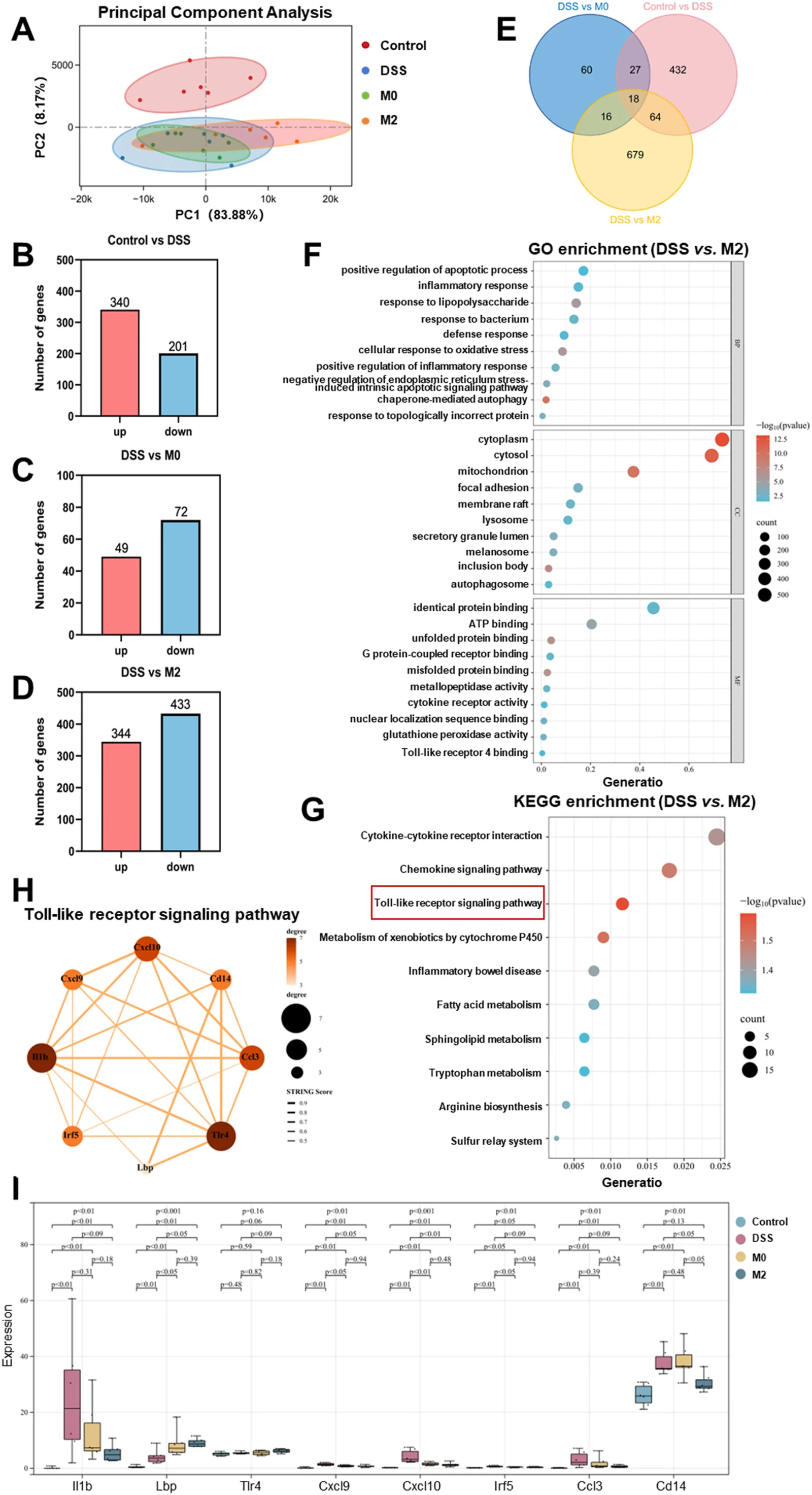
**Transcriptomic landscape of colonic epithelial cells following M2-MVs treatment. A,** Principal component analysis (PCA) of transcriptomes from Control, DSS, DSS + M0-MVs and DSS + M2-MVs groups. **B-D,** Numbers of differentially expressed genes (DEGs; upregulated in red, downregulated in blue) in the indicated pairwise comparisons. **E,** Venn diagram of DEG overlaps among the three comparisons. **F,** Gene Ontology (GO) enrichment bubble plot for DEGs between DSS and DSS + M2-MVs. Bubble size reflects gene count; colour indicates adjusted P-value. **G,** KEGG pathway enrichment bubble plot; the Toll-like receptor signaling pathway is highlighted. **H,** Interaction network of DEGs linked to the Toll-like receptor pathway. Node size, degree of connectivity; edge thickness, interaction strength. **I,** Expression distribution of selected inflammation- and chemotaxis-related genes (*Il1b, Lbp, Tlr4, Cxcl9, Cxcl10, Irf5, Ccl3, Cd14*). (For interpretation of the references to colour in this figure legend, the reader is referred to the Web version of this article.)

To elucidate the impact of M2-MVs on the transcriptional state of colonic epithelium during colitis, we performed RNA-sequencing (RNA-seq) on colonic epithelial cells from four treatment groups. Quality control assessments confirmed high sequencing quality and consistent expression profiles across samples, satisfying the requirements for subsequent differential expression and functional enrichment analyses (Figs. S6–S8). Principal component analysis (PCA) clearly distinguished the four groups, with good clustering of biological replicates (Fig. 5A). Heatmaps further revealed marked differences in mRNA expression profiles among treatment groups and robust intra-group clustering (Fig. S9). Analysis of the epithelial transcriptome suggested that, overall, DSS treatment evoked a clear transcriptional shift from Control. M2-MVs treatment shifted the transcriptome away from the DSS group towards the Control group, although it did not fully revert to the Control baseline. According to this finding it is possible that M2-MVs do not just reverse DSS-induced transcriptional changes but rather push epithelial cells towards a new steady-state programme characterized by decreased inflammation. M0-MVs, by contrast, had only modest effects. Differential expression analysis between the DSS and M2-MVs groups identified 777 differentially expressed genes (DEGs) (344 upregulated, 433 downregulated; Fig. 5B–D). Venn diagram comparisons indicated that while some of these DEGs overlapped with those identified in the Control versus DSS and DSS versus M0-MVs analyses, most were specific to the M2-MVs versus DSS comparison (Fig. 5E). This specificity further suggests that M2-MVs reshape the inflammatory transcriptional programme of the epithelium rather than merely antagonising it.

Gene Ontology (GO) enrichment analysis showed that these DEGs were primarily associated with inflammatory and host defence processes, including inflammatory response, response to lipopolysaccharide, innate immune response, defence response to bacterium and terms related to leukocyte chemotaxis (Fig. 5F). Consistent with these observations, KEGG pathway analysis revealed significant enrichment of the Toll-like receptor signaling pathway and the downstream NF-κB signaling cascade (Fig. 5G). In addition, GO and KEGG enrichment analyses of the NC (Control) versus DSS and DSS versus M0-MVs comparisons also showed changes in inflammatory responses, immune regulation and related signaling pathways, providing complementary information on the DSS-induced inflammatory phenotype and the characteristics of M0-MVs treatment (Figs. S10 and S11). The observed M2-MVs-associated transcriptomic changes were directly linked to the TLR4/MyD88/NF-κB signaling axis, a critical driver of mucosal inflammation, indicating that M2-MVs transcriptionally intercept pathogen-sensing and inflammatory amplification programmes in epithelial cells [33]. Among the inflammatory network hubs, changes in TLR4 mRNA levels across the three groups were relatively modest. However, network analysis identified TLR4 as a central, highly connected hub within the IL-1β-centred inflammatory interactome (Fig. 5H). We found that M2-MVs do not suppress downstream TLR4 signaling through transcriptional inhibition of TLR4 itself, but rather disrupt the TLR4 downstream effector network. Key inflammatory and immune-cell-recruitment nodes—including IL-1β, CXCL9, CXCL10 and CD14—showed elevated expression in the DSS and M0-MVs groups, whereas the M2-MVs group exhibited marked downregulation of these markers (Fig. 5I). IL-1β acts as a master amplifier of mucosal inflammation, disrupts epithelial tight junctions and synergises with the chemokines CXCL9 and CXCL10 to recruit neutrophils and other effector cells. CD14, as a co-receptor for TLR4, enhances the recognition of microbe-associated molecular patterns and sustains innate immune activation. By downregulating these hub transcripts, M2-MVs administration impairs the ability of epithelial cells to amplify and propagate inflammatory signals. Together, our transcriptomic data indicate that M2-MVs can rewire the inflammatory programme of colonic epithelial cells by downregulating a constellation of coupled mediators involved in the TLR4/MyD88/NF-κB axis. This rewiring restricts the amplification of inflammatory signals and chemokine-mediated immune cell recruitment at the transcriptional level [30]. These findings suggest that M2-MVs act as active agents that impose a barrier-protective and inflammation-resolving transcriptional identity on the epithelium, providing a mechanistic explanation for tissue-level barrier restoration and immune modulation.

### 2.6 M2-MVs suppress TLR4/MyD88/NF-κB to restore barrier integrity in colitis

**Fig. 6:**
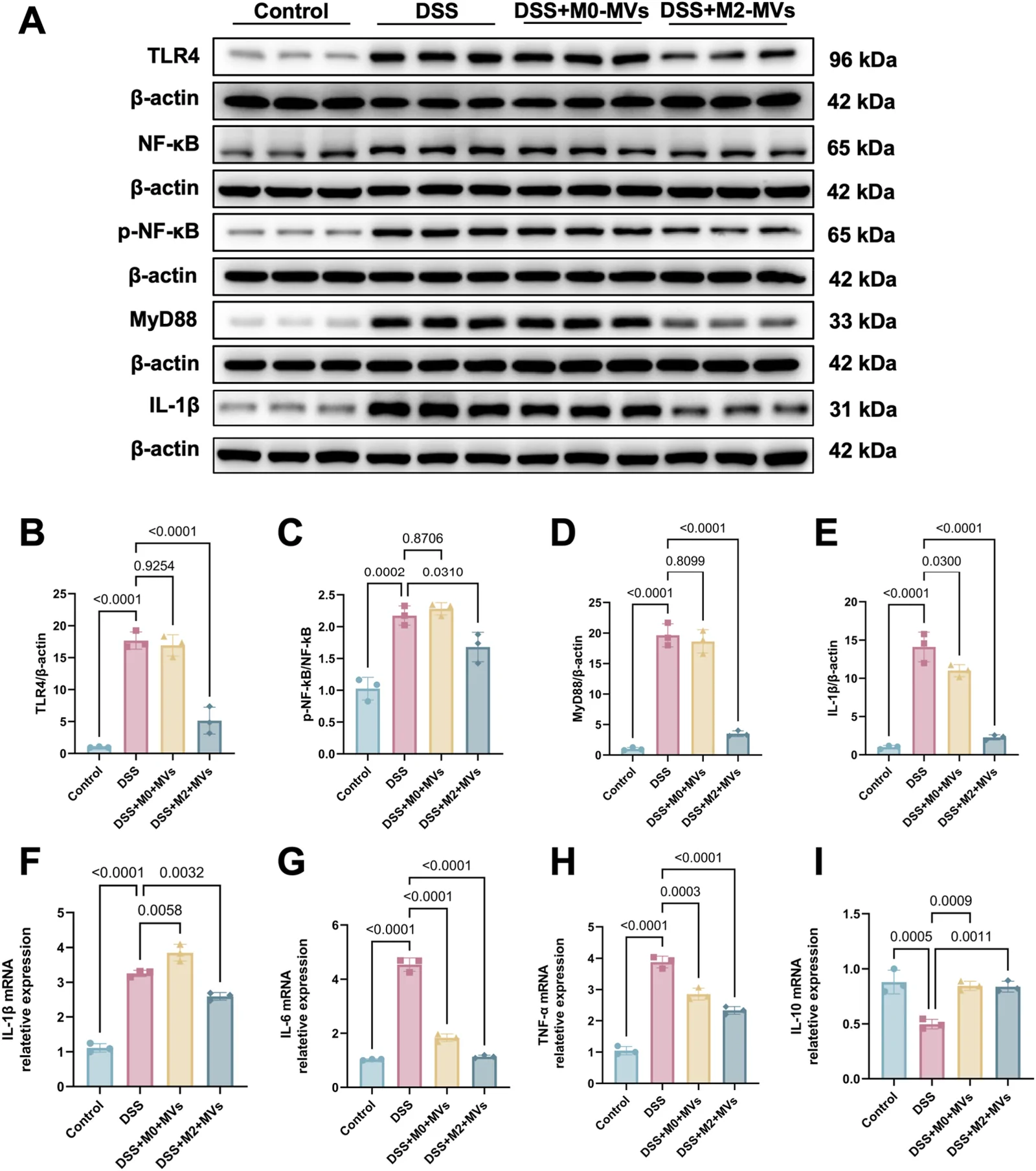
**M2-MVs suppress activation of the TLR4/MyD88/NF-κB pathway in colitic mice. A-E,** Western blot analysis of TLR4, p-NF-κB/NF-κB, MyD88, and IL-1β proteins in colon tissues from each group and the corresponding densitometric quantification. **F-I,** RT-qPCR analysis of the relative mRNA expression levels of IL-1β, IL-6, TNF-α, and IL-10 in colon tissues from each group. Data are presented as the mean ± standard deviation (SD), n = 3. Statistical significance is indicated in the figure.

**Fig. 7:**
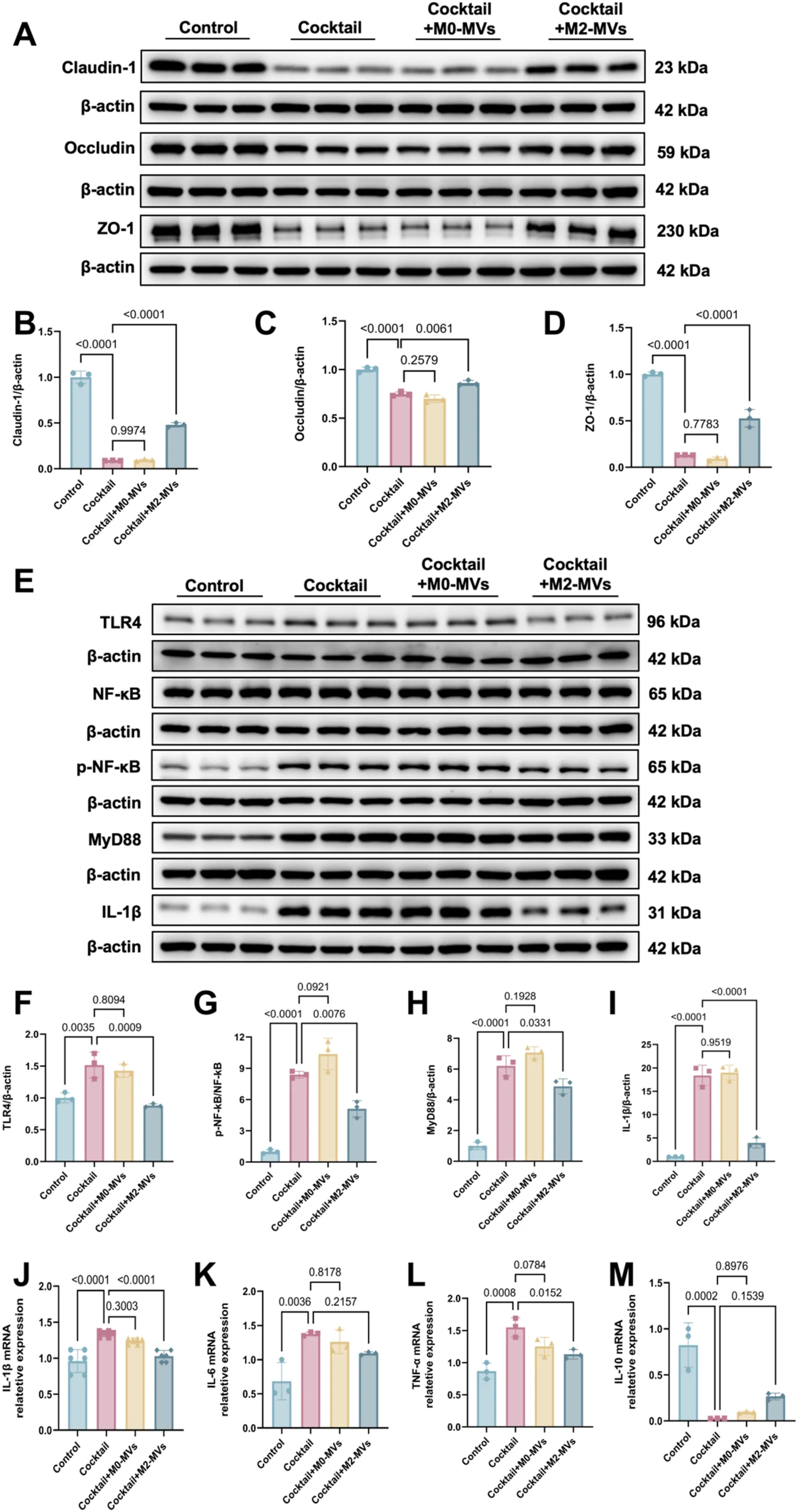
**M2-MVs alleviate barrier dysfunction and attenuate TLR4 signaling activation in inflamed colonic epithelial cells. A-D,** Western blot analysis of the protein expression levels of Claudin-1, Occludin, and ZO-1 in cells from each group and the corresponding densitometric quantification. **E-I,** Western blot analysis of the protein expression levels of TLR4, MyD88, p-NF-κB/NF-κB, and IL-1β in cells from each group and the corresponding densitometric quantification. **J-M,** RT-qPCR analysis of the relative mRNA expression levels of IL-1β, IL-6, TNF-α, and IL-10 in cells from each group. Data are presented as the mean ± standard deviation (SD), n = 3. Statistical significance is indicated in the figure.

Given the evidence that M2-MVs treatment reshapes the TLR4/MyD88/NF-κB inflammatory transcriptional network predominantly in colonic epithelial cells, we next sought to verify whether these effects also manifest at the protein and functional levels, and whether suppression of this cascade correlates with restoration of epithelial barrier integrity [34]. Analysis of colonic tissues showed that M2-MVs significantly reduced the protein abundance of TLR4, MyD88, phosphorylated NF-κB p65, and mature IL-1β in DSS-treated mice (Fig. 6A–E), whereas M0-MVs had no such inhibitory effect. The concurrent downregulation of TLR4 and its adaptor MyD88, together with reduced levels of the active phosphorylated form of the downstream transcription factor and decreased production of the transcriptional target IL-1β, indicate that M2-MVs block the pathway at multiple nodes. Furthermore, RT-qPCR analysis revealed that M2-MVs markedly reduced the mRNA levels of the pro-inflammatory cytokines IL-1β, IL-6 and TNF-α, while upregulating the anti-inflammatory cytokine IL-10 (Fig. 6F–I), suggesting a shift of the local cytokine milieu towards an anti-inflammatory profile. The strong concordance between our *in vivo* and *in vitro* observations supports the notion that suppression of epithelial inflammatory signaling is a core cell-intrinsic activity of M2-MVs, rather than an indirect consequence of systemic immunomodulation. In a model of human colonic epithelial NCM460 cells exposed to inflammatory cytokines, M2-MVs increased the protein expression of the tight-junction components Claudin-1, Occludin and ZO-1 (Fig. 7A–D). Concomitantly, M2-MVs decreased the protein levels of TLR4, MyD88, phosphorylated NF-κB p65 and IL-1β (Fig. 7E–I). The coordinated changes in gene expression (suppression of IL-1β, IL-6 and TNF-α, and enhancement of IL-10; Fig. 7J–M) were highly consistent with the *in vivo* findings, confirming that M2-MVs can directly regulate epithelial inflammatory machinery.

The suppression of inflammatory signals and restoration of the barrier can be viewed as parallel, mechanistically intertwined components of a self-reinforcing protective programme that may function as a bidirectional 'brake' on the intestinal inflammatory response. In the inflamed colon, where this vicious cycle is established, activation of the TLR4/MyD88/NF-κB axis leads to production of pro-inflammatory cytokines such as IL-1β and TNF-α. This in turn disrupts tight-junction integrity, increases paracellular permeability and facilitates translocation of luminal TLR4 ligands (including LPS and other microbe-associated molecular patterns), into the lamina propria, thereby perpetuating the cycle. M2-MVs apply the first brake by alleviating inflammation and inhibiting TLR4/MyD88/NF-κB signaling. Simultaneously, M2-MVs-mediated restoration of Claudin-1, Occludin, ZO-1 and MUC2 expression strengthens both the tight-junction and mucus barriers, limiting paracellular influx of TLR4 ligands and removing the stimulus for aberrant pathway activation-acting as a second, complementary brake. This integrated model illustrates how a single therapeutic intervention can concurrently achieve immunoregulation and mucosal repair without requiring distinct cargo components for each action. Instead, the cargo of M2-MVs-likely enriched in anti-inflammatory miRNAs, immunomodulatory cytokines and barrier-protective factors-may instruct epithelial cells to simultaneously dismantle the inflammatory signaling apparatus and establish a stable, inflammation-resistant epithelial phenotype by reconstructing junctional complexes. Our previous transcriptional profiling indicated that M2-MVs suppress an M1-like pro-inflammatory network centred on TLR4, IL-1β and chemokines involved in immune cell recruitment, providing a molecular framework for this bidirectional protective mechanism. Collectively, the TLR4/MyD88/NF-κB signaling axis is a critical therapeutic target of M2-MVs, and the inhibition of signaling together with barrier restoration are interdependent processes that mediate the protective action of M2-MVs against DSS-induced colitis.

### 2.7 M2-MVs remodel the colitic gut microecosystem

**Fig. 8:**
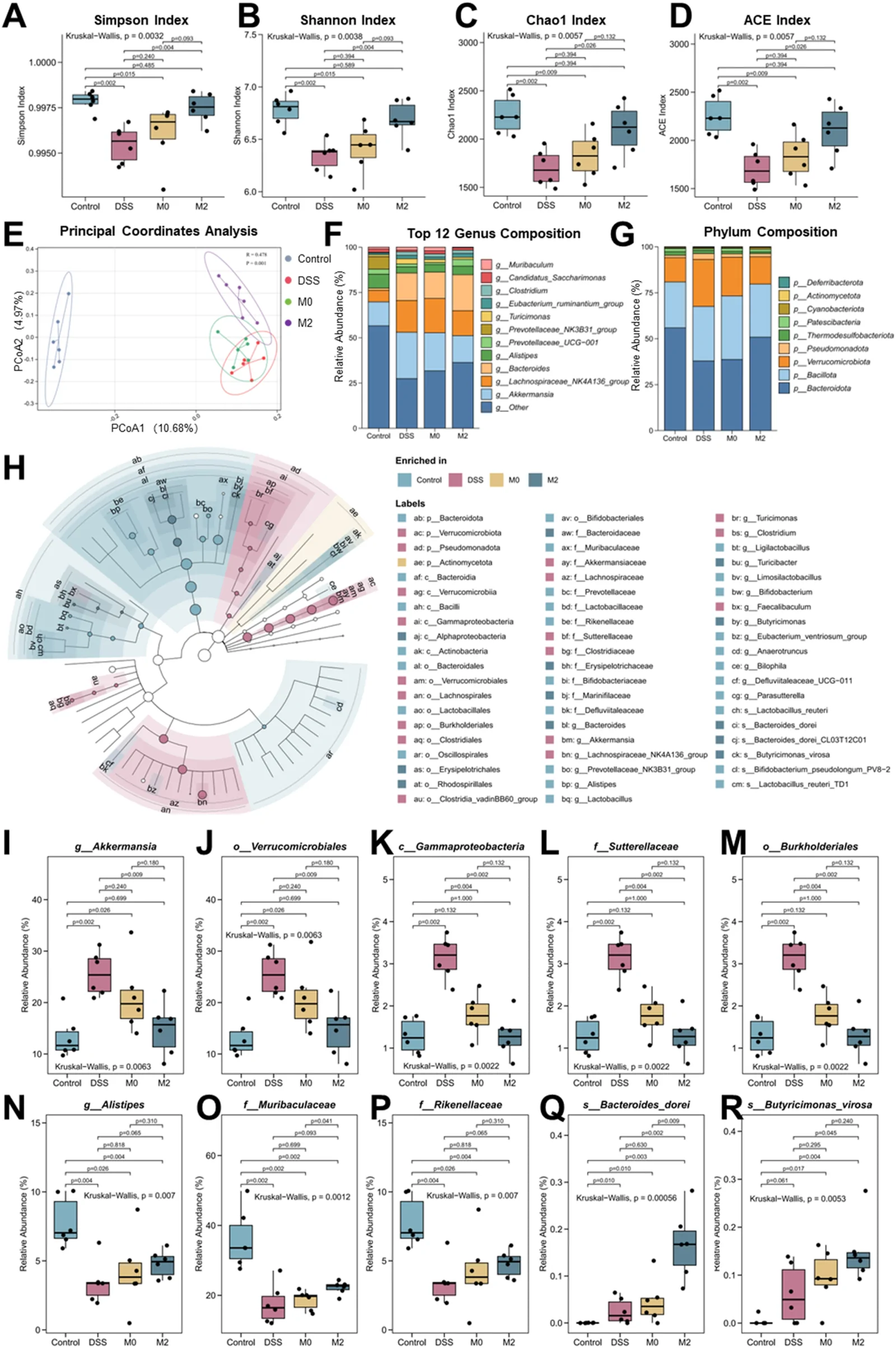
**M2-MVs alleviate DSS-induced gut microbiota dysbiosis. A-D,** Alpha diversity indices (Simpson, Shannon, Chao1, ACE) of the colonic microbiota. **E,** Beta diversity analysis based on principal coordinate analysis (PCoA). **F,** Stacked bar plot showing the relative abundance of the top 12 dominant genera in the gut microbiota of each group of mice. **G,** Stacked bar plot showing the relative abundance of gut microbiota at the phylum level in each group of mice. **H,** LEfSe cladogram illustrating differentially enriched phylogenetic lineages; coloured nodes denote taxa significantly enriched in the corresponding group. **I-R,** Quantitative relative abundances of the indicated differential genera. Each dot corresponds to one mouse (n = 6).

**Fig. 9:**
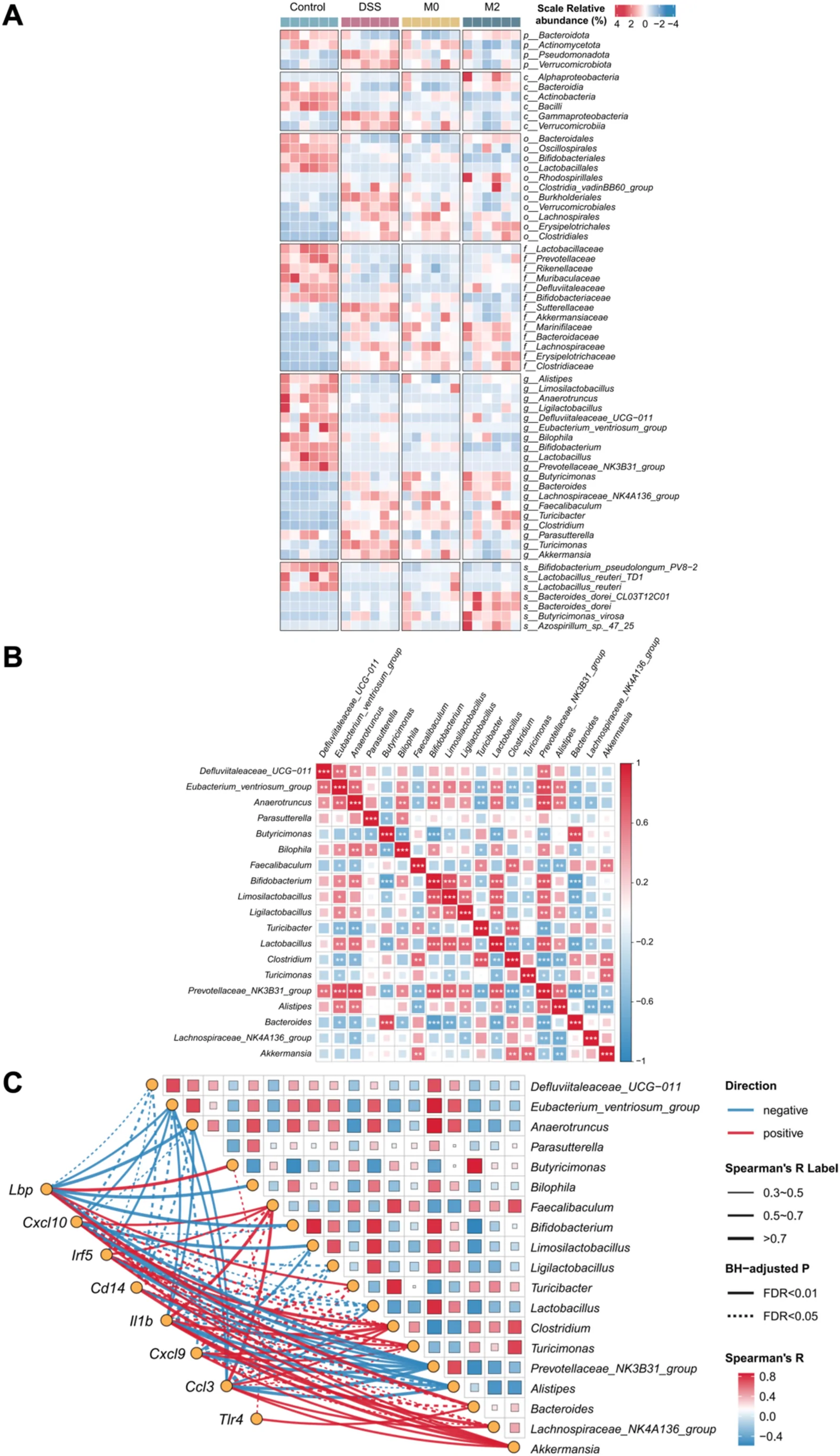
**Integrative analysis of microbial shifts and inflammation-related genes. A,** Heatmap depicting relative abundance of differential microbiota from phylum to species level across groups. Colours indicate Z-score-scaled relative abundance. **B,** Spearman correlation analysis among differential genera. Red indicates positive correlation; blue indicates negative correlation. The color intensity and square size reflect the strength of the correlation coefficient. Statistical significance is denoted as *, **, and *** for P < 0.05, 0.01, and 0.001, respectively. **C,** Spearman correlation network and combined heatmap linking inflammation-related genes (rows) with differential microbiota (columns). Red lines/boxes, positive correlation; blue, negative correlation. Solid and dashed lines indicate Benjamini–Hochberg corrected FDR < 0.01 and FDR < 0.05, respectively. (For interpretation of the references to colour in this figure legend, the reader is referred to the Web version of this article.)

To investigate the effect of M2-MVs on DSS-induced gut microbiota dysbiosis, we performed 16S rRNA gene sequencing on faecal samples from each group [35,36]. Rarefaction, rank-abundance and species accumulation curves indicated that the sequencing depth adequately captured the major microbial composition of the samples, satisfying the requirements for subsequent analyses (Figs. S12–S14). DSS treatment markedly reduced gut microbiota α-diversity, as reflected by decreased Simpson, Shannon, Chao1 and ACE indices. In contrast, M2-MVs treatment significantly restored these diversity metrics, whereas M0-MVs showed only limited recovery (Fig. 8A–D). Principal coordinate analysis revealed a clear separation in β-diversity between the DSS and control groups; the M2-MVs group deviated from the DSS group and partially shifted towards the control group, suggesting that M2-MVs not only alleviate the colitis phenotype but also promote gut microbial homeostasis (Fig. 8E). Furthermore, an ASV/OTU petal plot revealed shared as well as treatment-specific microbial features across groups, further supporting that DSS challenge and MVs interventions both influence microbial community composition (Fig. S15). Stacked bar plots at the phylum and genus levels showed marked differences in dominant microbial taxa among the four groups (Fig. 8F and G). Differential abundance analysis indicated that DSS induced substantial compositional abnormalities, and that MVs derived from differently polarized macrophages exerted distinct regulatory effects on the microbiota (Fig. 8H). In the DSS and M0-MVs groups, certain taxa such as *g_Akkermansia*, *o_Verrucomicrobiales*, *c_Gammaproteobacteria*, *f_Sutterellaceae*, and *o_Burkholderiales* were relatively more abundant, whereas their abundances were significantly lower in the control and M2-MVs groups (Fig. 8I–M). The findings indicate that M2-MVs inhibit the expansion of colitis-associated taxa. Earlier research has indicated that under inflammation, dysbiosis, and immune disturbance conditions that occur, *Gammaproteobacteria*, *Burkholderiales* and *Sutterellaceae* tend to bloom up. The decrease in numbers of these microorganisms after treatment with M2-MVs indicates that M2-MVs indeed have the capacity to promote damage repair and regulate inflammation and improve intestinal environment at the local level. A member of the order *Verrucomicrobiales*, *Akkermansia*, is a bacterium that could be beneficial in certain physiological and metabolic settings. In a colitis model induced by DSS, its presence was elevated initially, but robustly decreased after M2-MVs treatment. Thus, the biological significance of *Akkermansia* is highly context-dependent, particularly under inflammatory conditions. Injury to the gut mucosal lining may promote the enrichment of this genus by altering niches through changed mucus layer and altered microbiota. In comparison, the control and M2-MVs groups had higher proportions of *g_Alistipes* and *f_Muribaculaceae* and *f_Rikenellaceae,* but these were low in DSS and M0-MVs (Fig. 8N–P). These microbes are believed to play a role in gut homeostasis and are able to break down complex carbohydrates while generating metabolites helpful to other bacteria. After treatment with M2-MVs, the richness of these taxa seemed to elevate, but a statistical difference between DSS and M2-MVs groups did not exist. M2-MVs can exert a restorative impact on these taxa, although this effect is somewhat limited, likely because of treatment duration or inter-individual variability. Noteworthy is that the two species, *s_Bacteroides_dorei* and *s_Butyricimonas_virosa*, all showed a progressive increasing trend in different treatment groups. This indicates that M2-MVs not only help return the microbiota to a normal state. It may also lead to the establishment of a new microbial configuration (Fig. 8Q and R). Loss of microbial diversity, reduced community stability and functional imbalance are characteristic ecological features of DSS-induced colitis, consistent with the trends observed in this study. LEfSe analysis and heatmaps further demonstrated that DSS-induced compositional changes resulted in pronounced dysbiosis, whereas the microbial profile of the M2-MVs group more closely resembled that of the control group than that of the DSS group (Fig. 9A). Correlation analysis among differential genera revealed significant positive and negative associations, indicating that the microbial changes induced by DSS or MVs interventions are not isolated fluctuations of single genera but rather reflect co-ordinated community-level network remodeling (Fig. 9B). More importantly, significant correlations were observed between differential genera and the expression of colonic inflammation-related genes. Spearman correlation analysis showed that *Akkermansia* and certain other taxa were positively correlated with pro-inflammatory genes such as IL-1β, CXCL9, CXCL10 and CD14, whereas *Alistipes* was negatively correlated with these genes (Fig. 9C). These results indicate that DSS-induced microbiota dysbiosis is tightly linked to the host inflammatory response, and that M2-MVs may promote recovery of gut microecology and mucosal immune status by co-ordinately modulating the gut microbiota structure and the host inflammatory gene network. In summary, M2-MVs partially restore DSS-reduced gut microbiota α-diversity and species richness, suppress the aberrant expansion of inflammation-associated taxa, and promote the recovery of certain beneficial commensals. The correlations between differential genera and the expression of inflammatory genes further suggest that M2-MVs contribute to their protective effects against DSS-induced colitis through integrated regulation of the microbial community structure and the host inflammatory response network.

This integrated effect is consistent with recent multifunctional strategies for UC. An oral inulin-based nanomicelle combined H_2_S-mediated immunomodulation with prebiotic microbiota regulation, while a CO-releasing polyoxometalate nanozyme promoted mucosal immune regulation, microbiota homeostasis, and intestinal barrier repair.^39, 40^ These studies support the concept that simultaneous regulation of host inflammation, microbial homeostasis, and epithelial barrier function may provide greater therapeutic benefit than targeting an isolated pathological component. In this context, our findings extend this emerging therapeutic principle to a biologically derived, cell-free platform, demonstrating that M2-MVs can coordinate immunoregulation, barrier restoration, and gut microecosystem remodeling.

## 3 Conclusion

In experimental UC, M2-MVs attenuated disease manifestations while suppressing TLR4/MyD88/NF-κB signaling, improving tight-junction and mucus-barrier markers, and partially modulating DSS-associated microbial dysbiosis. The limited activity of M0-MVs supports an association between parental-cell polarization and vesicle function. These findings provide proof-of-concept for a cell-free, multimodal strategy but do not establish superiority to standard therapy or clinical readiness. Dose optimization, pharmacological-comparator studies, standardized manufacturing and quality control, comprehensive biosafety testing, identification of active cargo, and *in vivo* biodistribution analyses are required before translational development.

## CRediT authorship contribution statement

**Cuihua Qi:** Writing – original draft, Conceptualization. **Chaoyue Liang:** Writing – original draft, Conceptualization. **Sha Wang:** Investigation, Data curation, Conceptualization. **Hui Zhang:** Methodology, Formal analysis. **Han Wei:** Methodology, Formal analysis. **Xiaojun He:** Writing – review & editing, Funding acquisition. **Weigang Chen:** Writing – review & editing, Funding acquisition.

## Funding

This work was financially supported by Science and Technology Program of 10.13039/501100009967XPCC (2023ZD025), the 10.13039/501100004317Shihezi University Postdoctoral Research Project (336483), the 10.13039/501100004317Shihezi University First Affiliated Hospital Doctoral Fund (BS202104), and the Shihezi University School-Level Research Project (ZZZC202195).

## Declaration of competing interest

The authors declare that they have no known competing financial interests or personal relationships that could have appeared to influence the work reported in this paper.

## Data Availability

Data will be made available on request.
