# Supplementary material for: Engineered microvesicle-mediated multimodal therapy resolves ulcerative colitis via synergistic immunoregulation, barrier restoration, and microbiota remodeling
Source: Mater Today Bio. 2026 Aug 31;40:103623. doi: 10.1016/j.mtbio.2026.103623 (PMC13571736; doi:10.1016/j.mtbio.2026.103623)
Supplement: Supplementary file 1 [file mmc1.docx]

Supplementary Information

**Engineered microvesicle-mediated multimodal therapy resolves ulcerative colitis via synergistic immunoregulation, barrier restoration, and microbiota remodeling**

Cuihua Qi,^1,#^ Chaoyue Liang,^1,#^ Sha Wang,^1#^ Hui Zhang,^2^ Han Wei,^1^ Xiaojun He^3,*^ and Weigang Chen^1,*^

^1^Department of Gastroenterology, The First Affiliated Hospital of Shihezi University, Shihezi 832008 China

^2^College of Animal Science and Technology, Shihezi University, Shihezi 832003 China

^3^Department of Ophthalmology, Peking University First Hospital, Beijing 100034 China

All authors have approved the final version of this manuscript.

^#^These authors contributed equally to this work.

*Correspondence: [13579456959@126.com](mailto:13579456959@126.com) (W. Chen), [21394@pkufh.com](mailto:21394@pkufh.com) (X. He)

**Table of Contents**

**A.** Macrophage Culture and Polarization

**B.** Isolation and Purification of M0- and M2-Macrophage-Derived MVs

**C.** Animal Housing and Ethical Compliance

**D.** DSS-Induced Acute Colitis Model and Treatment Protocol

**E.** Colonic Epithelial Cell Isolation and Transcriptomic Profiling

**F.** Gut Microbiota Profiling by 16S rRNA Gene Sequencing

**G.** Inflammatory Cell Model Establishment and MVs Uptake Visualization

**H.** Quantitative Real-Time PCR for Gene Expression Analysis

**I.** Protein Extraction and Western Blot Analysis

**J.** Statistical Analysis and Reproducibility

**K.** Supplementary Figures

**A. Macrophage Culture and Polarization**

The macrophage cell line RAW264.7 (Shanghai Jinyuan Biotechnology, Cat#JY032) was cultured in high-glucose Dulbecco's Modified Eagle's Medium (DMEM, Servicebio, Cat#G4511) supplemented with 10% fetal bovine serum (FBS, Gibco, Cat#10091148) at 37°C in a humidified atmosphere containing 5% CO_2_. Upon reaching 70%–80% confluence, M2 polarization was induced by stimulating the cells with Mouse IL-4 Recombinant Protein (PeproTech, Cat#214-14) at 20 ng/mL and Mouse IL-13 Recombinant Protein (PeproTech, Cat#210-13) at 20 ng/mL for a duration of 24–48 hours. Concurrently, M1 polarization was induced by adding Lipopolysaccharides from Escherichia coli O55:B5 (LPS, Sigma-Aldrich, Cat#L2880) at 100 ng/mL and Mouse IFN-gamma Recombinant Protein (PeproTech, Cat#315-05) at 10 ng/mL for the same time period. Following induction, Western blotting was performed to assess the expression levels of macrophage polarization-associated markers. The M2 macrophage-associated markers CD206 and Arg1 were detected using MRC1/CD206 Antibody (Affinity Biosciences, Cat#DF4149) and ARG1 Antibody (Affinity Biosciences, Cat#DF6657), respectively, to verify M2 polarization. The M1 macrophage-associated markers CD86 and iNOS were detected using CD86 Antibody (Affinity Biosciences, Cat#DF6332) and iNOS Antibody (Affinity Biosciences, Cat#AF0199), respectively, to confirm M1 polarization.

**B.** Isolation and Purification of M0- and M2-Macrophage-Derived MVs

To prevent contamination from serum-derived exosomes, exosome-depleted serum was prepared prior to the experiment for the isolation of MVs. Ultracentrifugation was performed at 120,000 × g for 12 hours at 4°C to deplete the serum. The upper 70% of the serum was carefully collected without disturbing the pellet, sterilized by filtration through a 0.22 μm membrane, and stored at −20°C for future use. RAW264.7 cells that had not been polarized were designated as the M0 group, while cells treated with IL-4 and IL-13 for 24–48 hours were designated as the M2 group. The culture medium for both groups was then replaced with fresh medium containing exosome-depleted serum, and the cells were cultured for an additional 48 hours. Subsequently, the cell culture supernatants were collected to isolate MVs through differential centrifugation at 4°C. First, the supernatant was centrifuged at 2,000 × g for 20 minutes to remove cells and cellular debris. The resulting supernatant was then centrifuged at 16,500 × g for 75 minutes to pellet the MVs. The MVs pellets were resuspended in PBS (Servicebio, Cat#G4202) and subjected to a wash by centrifugation at 20,000 × g for 60 minutes. Finally, the purified MVs were resuspended in sterile PBS and stored at −80°C for long-term preservation.

**C.** Animal Housing and Ethical Compliance

Male C57BL/6JNifdc mice, obtained from Beijing Vital River Laboratory Animal Technology Co., Ltd., aged 6–8 weeks and weighing approximately 20 g, were housed in a specific pathogen-free (SPF) environment. The animal facility was maintained at a temperature of 24 ± 1°C, with relative humidity kept between 55 ± 5%. A 12-hour light/dark cycle was established, and all experimental procedures involving animals were approved by the Experimental Animal Ethics Committee of Shihezi University (Approval No. A2026-819). All experiments were conducted in strict compliance with relevant regulations concerning animal welfare and ethics, with all necessary efforts made to minimize the pain and suffering of the animals.

**D.** DSS-Induced Acute Colitis Model and Treatment Protocol

Twenty-four mice were randomly allocated to four experimental groups (n = 6 per group): the control group, DSS group, DSS + M0-MVs group, and DSS + M2-MVs group. Following a 7-day acclimatization period, all groups except the control group were administered 2.5% (w/v) Dextran sulfate sodium salt (DSS, APExBIO, Cat#B8205) in their drinking water ad libitum for 7 consecutive days to induce acute colitis. Mice in the control group received autoclaved drinking water throughout the experiment. On days 0, 2, 4, and 6, mice in the DSS + M0-MVs and DSS + M2-MVs groups received intraperitoneal injections of 50 µg of M0-MVs or M2-MVs in 200 µL of PBS, respectively, while mice in the DSS group were administered an equivalent volume of PBS at the same intervals. Samples were collected on day 8. Throughout the experiment, general condition, activity levels, stool consistency, and the presence of fecal blood were monitored daily, and body weights were recorded.

The exact number of animals contributing to each outcome is reported in the corresponding figure legend. Because colon tissues were allocated to histological examination, RNA sequencing, immunofluorescence, Western blotting, and RT-qPCR analyses, the available sample size differed among experimental endpoints. Western blotting and RT-qPCR analyses were performed using samples from three mice per group selected before outcome assessment. No outcome-based exclusions were performed.

**E.** Colonic Epithelial Cell Isolation and Transcriptomic Profiling

Colon tissues were longitudinally opened, washed with cold calcium- and magnesium-free Dulbecco’s PBS (DPBS, Servicebio, Cat#G4200), and cut into small fragments. The tissue fragments were pretreated with cold DPBS containing 1 μM dithiothreitol (DTT, Servicebio, Cat#GC205010), followed by incubation with 8 mM EDTA (prepared from a 0.5 M EDTA stock solution, Servicebio, Cat#G4051) at 4°C with gentle shaking for 75 minutes. Colonic epithelial cells were then released by vigorous shaking in cold DPBS, collected from the supernatant, pelleted by centrifugation at 800 × g for 5 minutes at 4°C, and stored at −80°C for short-term preservation, avoiding prolonged freezing. Total RNA was extracted using TRIzol reagent (Servicebio, Cat#G3013). RNA quantity and purity were assessed using a NanoDrop 2000 spectrophotometer, and RNA integrity was evaluated with an Agilent 2100 Bioanalyzer and the LabChip GX system. Samples meeting the quality standards were selected for library preparation using the VAHTS Universal V6 RNA-seq Library Preparation Kit for Illumina® (Vazyme, Cat#NR604-02), and subsequently sequenced on an Illumina NovaSeq X Plus platform. Enrichment analysis of Gene Ontology (GO) and Kyoto Encyclopedia of Genes and Genomes (KEGG) for the annotated differentially expressed genes was performed using the Phyper algorithm.

**F.** Gut Microbiota Profiling by 16S rRNA Gene Sequencing

The contents of the cecum and colon were collected, rapidly frozen in liquid nitrogen, and stored for downstream analysis. A magnetic bead extraction kit (TIANGEN, Cat#DP812) was employed to extract total genomic DNA. Fluorescence was utilized to determine DNA concentration, while gel electrophoresis or fragment analysis was performed to assess the quality of the amplified product. Primers targeting specific variable regions were used to amplify the bacterial 16S rRNA gene. The purified PCR products were then subjected to adapter ligation and library construction reactions. Following quality control, quantification, equimolar pooling, and purification, the libraries were sequenced on an Illumina NovaSeq 6000 platform.

**G.** Inflammatory Cell Model Establishment and MVs Uptake Visualization

NCM460 cells (Shanghai Jinyuan Biotechnology, Cat#JY817), a human normal colonic epithelial cell line, were cultured in high-glucose DMEM supplemented with 10% FBS at 37°C in a 5% CO2 atmosphere. Cells in the logarithmic growth phase demonstrating optimal condition and approximately 80% confluency were utilized for subsequent experiments. An in vitro inflammatory model was established by treating the cells with Recombinant Human IFN-gamma Protein (ABclonal, Cat#RP01038) at 30 ng/mL, Recombinant Human TNF-alpha Protein (ABclonal, Cat#RP00993) at 30 ng/mL, and Recombinant Human IL-1 beta Protein (ABclonal, Cat#RP00002) at 10 ng/mL for 48 hours.

For the MVs uptake assay, NCM460 cells in the logarithmic growth phase were seeded into confocal culture dishes and cultured until approximately 80% confluency was achieved. M0-MVs and M2-MVs were separately resuspended in PBS to a final volume of 200 µL and labeled with DiI (Shanghai Yuanye Bio-Technology, Cat#S25170) at a concentration of 5-10 µM for 30 minutes at 37°C in the dark. Post-labeling, an aliquot of the MVs suspension in PBS was centrifuged at 20,000 × g for 60 minutes to eliminate excess free dye. Subsequently, the DiI-labeled MVs were added to the culture dishes and co-incubated with NCM460 cells in the dark for 12 hours. Following incubation, the cells were washed three times with PBS and fixed with 4% paraformaldehyde for 15 minutes. Next, Hoechst 33342 (Shanghai Yuanye Bio-Technology, Cat#S19116) and DiO (Shanghai Yuanye Bio-Technology, Cat#S26181) were applied for 15-20 minutes to stain the nuclei, followed by a 15-20 minutes incubation at 37°C to stain the cell membranes. The samples were washed with PBS, mounted, and observed under a fluorescence microscope. DiI-labeled MVs were detected in the red channel, Hoechst-stained nuclei in the blue channel, and DiO-stained cell membranes in the green channel.

**H.** Quantitative Real-Time PCR for Gene Expression Analysis

Total RNA was extracted from tissue samples using the E.Z.N.A.® Total RNA Kit I (Omega Bio-tek, Cat#R6834) according to the manufacturer’s instructions. The concentration and purity of the RNA were subsequently assessed. A standardized amount of total RNA was reverse-transcribed into cDNA using the RevertAid First Strand cDNA Synthesis Kit (Thermo Fisher, Cat#K1622). Quantitative real-time PCR was then performed utilizing the synthesized cDNA and UltraSYBR Mixture (CWBIO, Cat#CW0957M). Gene-specific primers were synthesized by Shanghai Sangon Biotech Co., Ltd. The mRNA expression levels of target genes were normalized to an internal reference gene, and relative expression changes were calculated using the 2-ΔΔCt method. Assays were performed in triplicate wells, and experiments were independently repeated multiple times. The forward and reverse primer sequences are detailed in Table S3.

**I.** Protein Extraction and Western Blot Analysis

For sample preparation, total protein was extracted using RIPA Buffer (High) (Solarbio, Cat#R0010) supplemented with 1 mM phenylmethylsulfonyl fluoride (PMSF, Solarbio, Cat#P0100) and Phosphatase Inhibitor Cocktail I (MedChemExpress, Cat#HY-K0021). Protein concentrations were quantified using the Pierce™ BCA Protein Assay Kit (Thermo Fisher, Cat#23227). Protein samples were mixed with a 5× SDS loading buffer (Biosharp, Cat#BL530A) and denatured by heating at 100°C for 10 minutes. Subsequently, proteins were separated via 10% SDS-PAGE (Servicebio, Cat#G2177) and transferred onto polyvinylidene fluoride (PVDF) membranes (Millipore, Cat#IPVH00010). The membranes were blocked with Bovine Serum Albumin (BSA, Servicebio, Cat#GC305010) for 1 hour at room temperature, followed by overnight incubation at 4°C with the following primary antibodies: Anti-TLR4 Antibody for mouse samples (Boster Biological Technology, Cat#A00017-3), Anti-TLR4 Antibody for human samples (Boster Biological Technology, Cat#A00017-2), Anti-NF-κB p65/RELA Antibody (Boster Biological Technology, Cat#A00284-1), Phospho-NF-kB p65 (S536) Recombinant Rabbit Monoclonal Antibody (HUABIO, Cat#HA723223), Anti-MYD88 Antibody (Boster Biological Technology, Cat#PB9148), Claudin-1 Antibody (Affinity Bio, Cat#AF0127), Occludin Antibody (Boster Biological Technology, Cat#A01246-4), ZO-1 Polyclonal Antibody (Proteintech, Cat#21773-1-AP), and β-Actin Rabbit mAb (ABclonal, Cat#AC026). The following day, the membranes were washed three times (5 minutes each) with Tris-buffered saline (TBS, Servicebio, Cat#G0001) containing Tween 20 (Merck, Cat#P1379). The membranes were then incubated with corresponding secondary antibodies for 1 hour at room temperature. Protein bands were developed using an ultra-sensitive ECL substrate (Biosharp, Cat#BL5965B) and visualized using an imaging system.

**J.** Statistical Analysis and Reproducibility

Image quantification was performed using ImageJ software. Statistical analyses were conducted using SPSS version 22.0 and GraphPad Prism version 9.5. Data are presented as the mean ± standard deviation (SD). For in vivo experiments, the individual animal was considered the experimental unit. Technical replicates were averaged to generate one value for each biological replicate and were not treated as independent observations. Unless otherwise indicated, all in vitro experiments were performed with a minimum of three independent biological replicates, and the sample sizes for in vivo experiments are specified in the corresponding figure legends. Comparisons among multiple groups were performed using one-way analysis of variance (ANOVA), followed by Tukey’s honestly significant difference (HSD) post hoc test for pairwise comparisons. The statistical test used, sample size or number of independent experiments, and definitions of statistical significance are provided in the corresponding figure legends. A P value < 0.05 was considered statistically significant.

**K.** Supplementary Figures

**Figure S1.** Illustration of the MVs pellet.

**Figure S2.** The zeta potential characterization of MVs derived from M0 and M2 macrophages was conducted using a nanoparticle size and zeta potential analyzer. The zeta potentials of MVs derived from unpolarized macrophages (M0-MVs) and those from M2-polarized macrophages (M2-MVs) were measured. Data are presented as the mean ± SD from n = 3 independent MVs preparations.

**Figure S3.** Fecal color and anal bleeding in mice from different treatment groups. A, Fecal appearance of mice in the Control, DSS, DSS+M0-MVs, and DSS+M2-MVs groups. B, Anal bleeding (perianal bloodstaining) in mice from the Control, DSS, DSS+M0-MVs, and DSS+M2-MVs groups.

**Figure S4.** Effects of macrophage-derived MVs on cell viability. Cell viability was assessed using the cell counting kit-8 (CCK-8) assay. Cells were treated with various concentrations (0, 5, 10, 15, and 20 ng/mL) of M0 macrophage-derived MVs (M0-MVs) and M2 macrophage-derived MVs (M2-MVs) for 24 hours (top) and 48 hours (bottom). Based on the viability results, a moderate concentration of 15 ng/mL was selected as the optimal working concentration for subsequent experiments.

**Figure S5.** Quantitative analysis of immunofluorescence staining for intestinal barrier-related markers. Bar graphs present the positive staining area ratios for Claudin-1, Occludin, ZO-1, and MUC2 across the Control, DSS, DSS+M0-MVs, and DSS+M2-MVs groups.

**Figure S6.** Sequencing quality assessment of the RNA-seq data. A, Distribution of base quality scores along the read positions. B, Distribution of base composition (percentage of bases) along the read positions.

**Figure S7.** Evaluation of read distribution, sequencing saturation, and mRNA fragmentation randomness. A, Distribution of mapped reads across genomic regions. B, Sequencing saturation analysis. C, Randomness assessment of mRNA fragmentation.

**Figure S8.** Boxplots of expression distribution across samples. The x-axis represents the samples, and the y-axis displays log2(FPKM+1), illustrating the consistency of gene expression distributions among the different samples.

**Figure S9.** Heatmaps illustrating the expression intensity of all 23,095 detected mRNAs versus the top 500 differentially expressed mRNAs. A, Overall expression patterns of 23,095 mRNAs across the different samples. B, Expression patterns of the top 500 mRNAs exhibiting the most prominent expression changes. The x-axis represents the samples, and the y-axis represents the mRNAs. Colors indicate relative gene expression levels. These heatmaps illustrate the overall differences and clustering patterns of the mRNA expression profiles among the different groups.

**Figure S10.** GO enrichment analysis of differentially expressed genes in different comparison groups. The figure shows the top 20 enriched GO terms of differentially expressed genes in each comparison, classified into Biological Process (BP), Cellular Component (CC), and Molecular Function (MF). A, Control vs. DSS comparison, showing the GO enrichment results of genes upregulated and downregulated in the DSS group. B, DSS vs. M0 comparison, showing the GO enrichment results of genes upregulated and downregulated in the M0 group. The x-axis indicates the GO term names, and the y-axis indicates the number of differentially expressed genes enriched in each term (Count).

**Figure S11.** KEGG enrichment analysis of differentially expressed genes in different comparison groups. The figure shows 20 enriched KEGG pathways of differentially expressed genes in each comparison, classified by functional category. A, Control vs. DSS comparison, showing the KEGG enrichment results of genes upregulated and downregulated in the DSS group. B, DSS vs. M0 comparison, showing the KEGG enrichment results of genes upregulated and downregulated in the M0 group. The x-axis indicates the number of differentially expressed genes enriched in each pathway (Count), and the y-axis indicates the KEGG pathway names.

**Figure S12.** Rarefaction curves based on 16S sequencing data. The x-axis represents the number of randomly sampled sequencing reads, and the y-axis represents the number of detected OTUs/ASVs at the corresponding sequencing depth. Different colored curves represent different samples or groups. As the number of sequencing reads increases, the number of detected OTUs/ASVs gradually approaches a plateau, indicating that the current sequencing depth is sufficient to capture the microbial diversity of the samples.

**Figure S13.** Rank abundance curves of OTUs/ASVs. The x-axis indicates the OTU/ASV rank, and the y-axis indicates relative abundance. Different colored curves represent different samples, illustrating species richness and evenness within each sample.

**Figure S14.** Species accumulation curve. The x-axis represents the number of samples, and the y-axis represents the number of species detected after sampling. The blue boxplots form the accumulation curve, while the red boxplots form the shared-species curve, reflecting changes in species richness and the number of shared species among samples as the sample size increases.

**Figure S15.** Petal diagram of shared and unique ASV/OTU numbers among different groups. Different colored regions represent different groups. The central region indicates the number of ASVs/OTUs shared among all groups, while the non-overlapping regions indicate the numbers of unique ASVs/OTUs in each respective group.

**Table S1.** Evaluation of macroscopic scores.^1^

| Score | Criteria for colonic damage |
| --- | --- |
| 0 | No visible damage |
| 1 | Hyperemia without ulceration |
| 2 | Hyperemia with thickening of the colonic wall, but without ulceration |
| 3 | A single ulcerative lesion without colonic wall thickening |
| 4 | Two or more ulcerative lesions |
| 5 | Inflammatory lesion or severe damage extending approximately 0.5 cm |
| 6–10 | Inflammatory lesion or severe damage extending approximately 1 cm |

**Table S2.** Histopathological scores.^2^

| Score | Extent of inflam-  mation | Neutrophil and lympho-  histiocytic infiltration | Crypt damage | Crypt abscess | Submucosal edema | Goblet cell loss | Reactive epithelial hyper-  plasia |
| --- | --- | --- | --- | --- | --- | --- | --- |
| 0 | None | None | None | None | None | None | None |
| 1 | Mucosa | Focal | Basal one-third of crypts affected | Focal | Focal | Focal | Focal |
| 2 | Mucosa and sub-  mucosa | Multifocal | Basal two-thirds of crypts affected | Multi-  focal | Multifocal | Multi-  focal | Multi-  focal |
| 3 | Mucosa, sub-  mucosa, and muscular layer | Diffuse | Entire crypt destruction | — | Diffuse | Diffuse | Diffuse |
| 4 | Trans-  mural | — | Crypt destruction with ulceration | — | — | — | — |

**Table S3.** Primer sequences for RT-qPCR.

| **Gene** | **Forward sequence (5′→3′)** | **Reverse sequence (5′→3′)** |
| --- | --- | --- |
| Mouse CD86 | GGTGGCCTTTTTGACACTCTC | TGAGGTAGAGGTAGGAGGATCTT |
| Mouse iNOS | GTTCTCAGCCCAACAATACAAGA | GTGGACGGGTCGATGTCAC |
| Mouse CD206 | CTCTGTTCAGCTATTGGACGC | CGGAATTTCTGGGATTCAGCTTC |
| Mouse ARG1 | CTCCAAGCCAAAGTCCTTAGAG | AGGAGCTGTCATTAGGGACATC |
| Mouse IL-1β | GCAACTGTTCCTGAACTCAACT | ATCTTTTGGGGTCCGTCAACT |
| Mouse IL-6 | TAGTCCTTCCTACCCCAATTTCC | TTGGTCCTTAGCCACTCCTTC |
| Mouse TNF-α | ACTGAACTTCGGGGTGATCG | CCACTTGGTGGTTTGTGAGTG |
| Mouse IL-10 | GCTCTTACTGACTGGCATGAG | CGCAGCTCTAGGAGCATGTG |
| Mouse β-actin | GGCTGTATTCCCCTCCATCG | CCAGTTGGTAACAATGCCATGT |
| Human IL-1β | TGAGCTCGCCAGTGAAATGA | TGAAGCCCTTGCTGTAGTGG |
| Human IL-6 | CCGGGAACGAAAGAGAAGCTC | ACCGAAGGCGCTTGTGGAG |
| Human TNF-α | CCCTCACACTCAGATCATCTTCT | GCTACGACGTGGGCTACAG |
| Human IL-10 | GACTTTAAGGGTTACCTGGGTTG | TCACATGCGCCTTGATGTCTG |
| Human GAPDH | AATGGGCAGCCGTTAGGAAA | GCCCAATACGACCAAATCAGAG |

**References**

1.	Luo, R. et al. Oral microsphere formulation of M2 macrophage-mimetic Janus nanomotor for targeted therapy of ulcerative colitis. *Sci. Adv.* 10 (2024) eado6798.

2.	Zhang, H. et al. Oncometabolite 5-IP7 inhibits inositol 5-phosphatase to license E-cadherin endocytosis. *Nat. Chem. Biol.* 22 (2026) 293-306.
